# Modelling monthly pan evaporation utilising Random Forest and deep learning algorithms

**DOI:** 10.1038/s41598-022-17263-3

**Published:** 2022-07-30

**Authors:** Mustafa Abed, Monzur Alam Imteaz, Ali Najah Ahmed, Yuk Feng Huang

**Affiliations:** 1 Department of Civil and Construction Engineering, Swinburne University of Technology, Hawthorn, Melbourne, VIC 3122 Australia; 2 Department of Civil Engineering, College of Engineering, Universiti Tenaga Nasional (UNITEN), 43000 Selangor, Malaysia; 3 Department of Civil Engineering, Lee Kong Chian Faculty of Engineering and Science, Universiti Tunku Abdul Rahman, Selangor, Malaysia

**Keywords:** Hydrology, Engineering

## Abstract

Evaporation is the primary aspect causing water loss in the hydrological cycle; therefore, water loss must be precisely measured. Evaporation is an intricate nonlinear process occurring as a result of several climatic aspects. The purpose of this research is to assess the feasibility of using Random Forest (RF) and two deep learning techniques, namely convolutional neural network (CNN), and deep neural network (DNN) to accurately estimate monthly pan evaporation rates. Month-based weather data gathered from four Malaysian weather stations during the 2000–2019 timeframe was used to train and evaluate the models. Several input attributes (predictor variables) were investigated to select the most suitable variables for machine learning models. Every approach was tested with several models, each with a different set of model aspects and input parameter combinations. The formulated ML approaches were benchmarked against two commonly used empirical methods: Stephens & Stewart and Thornthwaite. Model outcomes were assessed using standard statistical measures to determine their effectiveness in predicting evaporation. The results indicated that the three ML models developed in the study performed better than empirical models and could significantly improve the precision of monthly Ep estimates even with the identical input sets. The performance assessment metrics also show that the formulated CNN approach was acceptable for modelling monthly water loss due to evaporation with a higher degree of accuracy than other ML frameworks explored in this study. In addition, the CNN framework outperformed other AI techniques evaluated for the same areas using identical data inputs. The investigation’s findings in relation to the various performance criteria show that the proposed CNN model is capable of capturing the highly non-linearity of evaporation and could be regarded as an effective tool to predict evaporation.

## Introduction

Evaporation is among the vital aspects that have a pivotal role in regulating the hydrological cycle; forecasting evaporation loss is critically important for water management, irrigation planning, and agricultural models^1–4^. Increased evaporation rate is a significant global warming indicator^5^. Therefore, recording evaporation patterns is critical for monitoring and handling water resources^6^. Evaporation causes significant water loss, impacting water levels in lakes and reservoirs, affecting the water budget. Therefore, before designing irrigation systems and implementing water resource strategies, evaporation losses must be estimated^7^. Reliable evaporation forecasting is critical for hydrological and water resources, enhancement of water use, and water balance. Vapour pressure difference and heat availability affect evaporation rates; these determining factors are affected by meteorological aspects, such as solar radiation, humidity levels, wind speed, air temperature, and air pressure^8–10^. Such factors are also deeply associated with other characteristics like geographical location, seasonal influence, climate type, and time of day. Hence, evaporation is a complex phenomenon with extremely non-linear characteristics.

Evaporation estimation is conducted using indirect and direct techniques, including energy balance, water balance, mass transfer, Penman method, and evaporation pan^11^. The evaporation pan is an extensively used apparatus because it is inexpensive and easy to use^12^. Nevertheless, this is an energy-intensive process affected by numerous meteorological aspects like wind speed and vapour pressure. Moreover, pan evaporimeters cannot be deployed at every required location, specifically those where instruments cannot be installed or managed^13^. Indirect techniques comprise evaporation determination using meteorological information and physical concepts like volume and energy conservation that require precise adjustment based on climate. Accurately determining such meteorological variables is challenging and requires advanced instruments and skilled labour^14^. However, it is known that such techniques cannot offer reliable evaporation data because of intrinsic complications and the non-linear nature of the evaporation process. Considering the inadequate performance levels, such techniques have prompted scientists to develop alternative methods for determining evaporation levels^15^.

### Literature review

Recently, AI techniques like ANN, M5 model tree (MT), support vector machines (SVM), adaptive neuro-fuzzy inference system (ANFIS), extreme learning machine (ELM), and gene expression programming (GEP) have been used to handle different water engineering and environmental issues^16–21^. Such AI techniques are simpler, more robust and can model complex non-linear processes without significant problems^13,22,23^. Extensive research has been conducted about using AI to forecast different hydrological parameters^24^. Researchers assert that ANN frameworks provide better forecasts than conventional methods. For example, Castellano-Méndez et al.^25^ contrasted the Box & Jenkins approach with ANN; the latter provides better runoff simulation performance in terms of precision.

Concerning evaporation forecasting and considering the challenges of practical and conceptual measurement techniques discussed above, several works have been performed using ML approaches with several optimisation works for forecasting pan evaporation^26,27^. They offered specific distinct machine learning approaches for the problem using different input sets concerning existing climatic attributes like wind speed, temperature, humidity, vapour pressure, solar radiation, and sunshine^28,29^. Keskin and Terzi^30^ used ANN and Penman models to develop evaporation models. They employed several meteorological aspects as ANN inputs. These researchers indicated that ANNs were superior to the Penman approach for evaporation forecasting. Kişi ^31^ formulated evolutionary neural networks to estimate pan evaporation for monthly timescales. The results indicated that the formulated models provided better accuracy than empirical methods. Deo et al.^32^ researched monthly water loss due to evaporation; they used three machine learning techniques: Relevance Vector Machine (RVM), Extreme Learning Machine (ELM), and Multivariate Adaptive Regression Spline (MARS). Meteorological aspects were employed as independent variables, and RVM was found to be the most effective approach among these. Sudheer et al. ^22^ formulated an ANN approach for modelling daily evaporation and mentioned that ANN frameworks could be effectively employed to forecast evaporation using climate data. Falamarzi et al. ^33^ evaluated ANN and wavelet ANN use to forecast daily evaporation. They employed temperature and wind speed data as model inputs. The results indicated that the two frameworks estimated evaporation precisely. Wang et al. ^34^ estimated daily evaporation using multivariate adaptive regression spline (MARS), least-square support vector regression (LSSVR), fuzzy genetic (FG), multiple linear regression (MLR), and M5 model tree (M5Tree) for eight locations near the Dongting Lake basin in China. The outcomes indicated that FG and LSSVR offer better performance and estimate evaporation with high accuracy. Malik et al. ^35^ estimated monthly Ep in the central Himalayan region in India using radial basis neural network (RBNN), multilayers perceptron neural network (MLPNN), self-organising map neural network (SOMNN), and co-active neuro-fuzzy inference system (CANFIS). The appropriate input set was selected using the Gamma test. The researchers found that the AI-powered technique could be employed for precise evaporation prediction. Tezel and Buyukyildiz ^36^ studied the applicability of MLP, RBFN, and e-support vector regression (SVR) using numerous training methods. When scaled conjugate gradient (SCG) learning was used for ANN and SVR approaches, performance was higher than empirical approaches.

Tree-based Machine learning approaches like RF have been used extensively for water and other environmental modelling during the past decade to estimate aspects like groundwater levels, streamflow, solar radiation, soil moisture, evaporation (e.g., pan and potential evapotranspiration), and suspended sediment. Such methods are relatively straightforward but potent approaches for pattern or trend detection ^37,38^; moreover, they offer more computationally efficient for relatively large datasets than other machine learning techniques^39^. Francke et al. ^40^ employed quantile regression forests (QRF) to estimate suspended sediment concentration for four sub-catchment areas in Spain. QRF results were contrasted against RF, generalised linear model, and the traditional sediment rating curve. The researchers found that QRF and RF are extremely flexible techniques that successfully modelled sediment dynamics. Feng et al. ^41^ employed the RF framework to predict daily evaporation in southwest China and contrasted it against the GRNN approach. The outcomes indicated that GRNN and RF approach provided acceptable results concerning daily evaporation; RF was marginally superior to GRNN. Recently, DL methods have been used in the machine learning domain and demonstrated success for data evaluation for natural processes, attracting attention concerning time series forecasts ^42^. Deep learning is a recent development in the ML paradigm that evolved from near-human to super-human performance for several engineering scenarios. In this class, forecasts are impacted by prior system characteristics; therefore, they may be used for regression and classification problems. Evaporation is intrinsically complex, dynamic, and non-linear; thus, the adaptive evaporation estimation framework must process nonlinear properties. Of the many ANN models specified in the literature, DL can process higher-order non-linear characteristics with better performance concerning time series data and its intrinsic properties for extended durations to enhance forecasting performance^43^. Convolutional Neural Network (CNN) has garnered extensive attention in the deep learning technique domain due to its use in several domains like object recognition^44^, time series categorisation^45^, audio signal classification^46^, and robotic visual and haptic data classification^47^, and weather forecasting^48^. In addition, in the noisy time series context, convolutional networks also reduce data noise and identify useful patterns by building hierarchical structures^49^. It must be noted that several academicians have used CNN for numerous time series prediction fields like solar energy forecast, electrical load estimation, and other scenarios.

The literature review confirmed that using ANN with appropriate learning methods can suitably model evaporation for numerous locations with superior results than relatively complex conventional approaches^50^. However, identifying and devising efficacious, reliable, and generalised evaporation estimation techniques is still challenging for researchers because of the intricate and non-linear nature of the evaporation process. Among the diverse ANN methods used in the recent past, the cutting-edge DL approach offers immense potential for prediction problems and has outperformed more complex methods. Because prediction is a nonlinear task, the adaptive framework for prediction ought to be nonlinear as well. With the success of DL, the CNN has become extremely advantageous for extracting characteristics from time-series data signals and thus for classification and prediction. The most important aspect of this approach is that it identifies implied recurrent sequences from the series. Moreover, such networks automatically use data to identify features without additional training or prior information. The CNN is powerful in capturing high nonlinear features among the various DL structures reported in the literature. Hence, in the current study, CNN was selected for the monthly pan evaporation forecast.

### Objectives

This study is intended to assess the predictability and applicability of the CNN model in accurately estimating monthly Ep rates in four Malaysian regions using weather data for the period 2000–2019. The performance of the CNN model is compared with that of the RF as a powerful tree-based technique and with the DNN model. The models’ prediction accuracy is explored under various input combination scenarios. The proposed ML frameworks are contrasted against two widely used empirical methods, namely Thornthwaite and Stephens & Stewart, under identical input combinations. The model’s efficiency values are assessed and analysed using standard statistical performance metrics to determine their use in predicting evaporation levels. Furthermore, sufficient analysis would be performed in this study to demonstrate the reliability of the CNN model, with the goal of developing a dependable model for predicting evaporation, which is essential, specifically in water resource management and agricultural planning.

## Study area and data

### Study area

**Figure 1:**
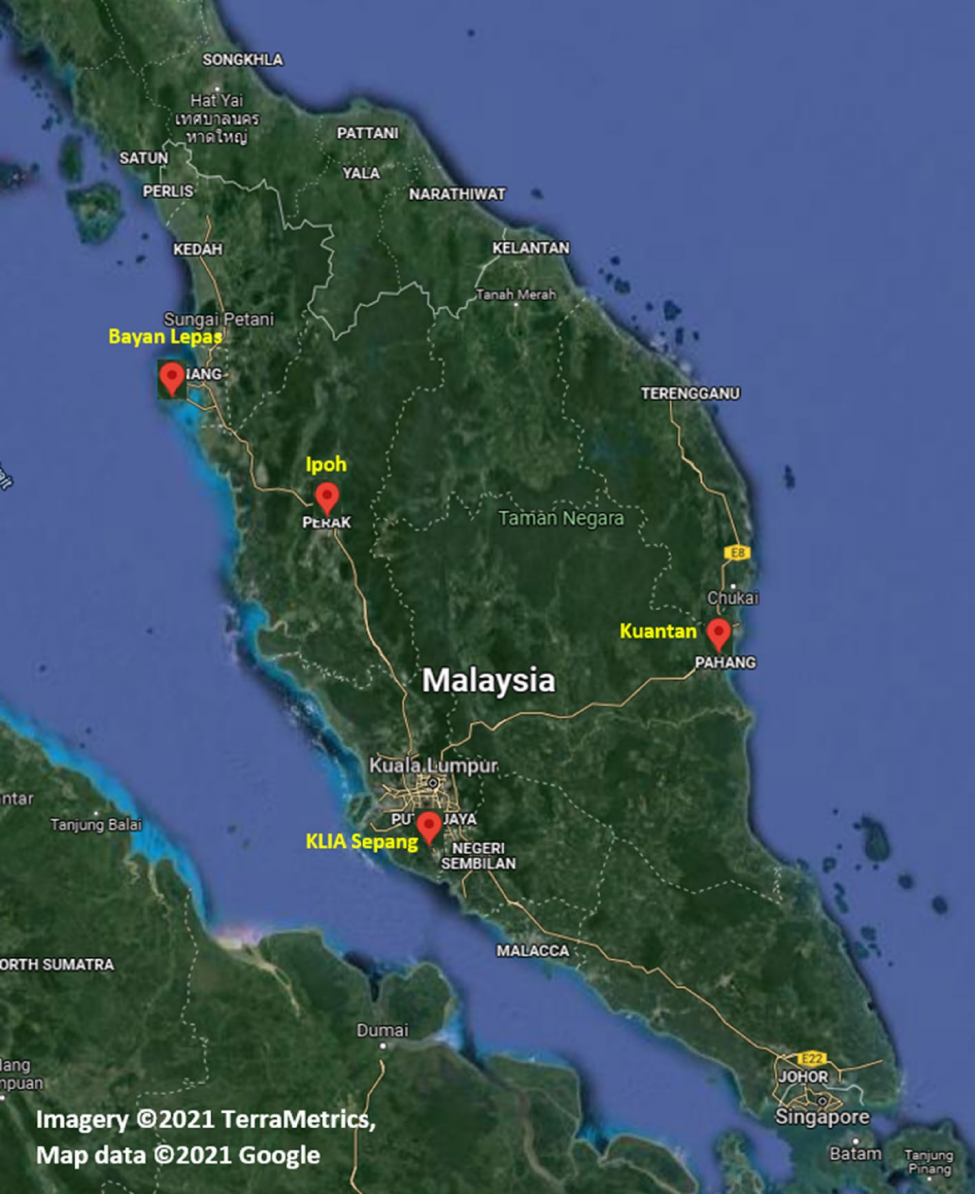
Location of case study [Imagery ©2021 TerraMetrics, Map data ©2021 Google].

Malaysia is in the tropical region and receives ample rainfall. Nevertheless, development has spiked water requirements. Additionally, climate change has extended the dry season and increased the evaporation rate from reservoirs. Many consider drought a very intricate but poorly understood natural calamity, impacting people more than other hazards^51^; hence, predicting evaporation is vital. Therefore, this research, which aims to develop accurate models for predicting Ep, is extremely important, particularly in water resource management and agriculture. The climate monthly data from four meteorological stations situated in Bayan Lepas (longitude 100° 16′ E, latitude 5° 18′ N, elevation 2.5 m), Ipoh (longitude 101° 06′ E, latitude 4° 34′ N, elevation 40.1 m), KLIA Sepang (longitude 101° 42′ E, latitude 2° 44′ N, elevation 16.1 m), and Kuantan (longitude 103° 13′ E, latitude 3° 46′ N, elevation 15.2 m), managed by the MMD (Malaysian Meteorological Department), are utilised to calibrate and corroborate the recommended predictive models. Figure 1 depicts Malaysia’s map where the four stations are situated; Google Maps were used to create this map depicting the studied region.

### Data description

**Figure 2:**
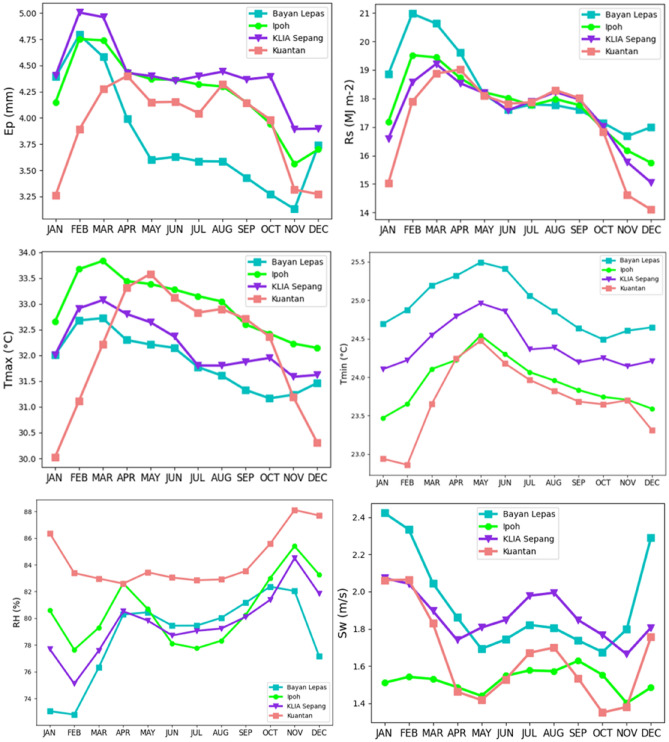
Monthly variations of Ep and related meteorological parameters used in this study.

The propositioned predictive models were built using seven meteorological indicators that include T_max_, T_min_, T_a_, RH, S_w_, R_s_, and E_p_. The data set consisted 19 years of day-to-day reports from 2000 to 2019. The statistical parameters recorded every month pertaining to the quantified meteorological data for the four above-mentioned stations are listed in Table 1. Moreover, Fig. 2 illustrates the monthly variation of every weather parameter for the duration 2000 to 2019.

**Table 1:** Various meteorological variables and their descriptive statistics.

Station	Dataset	Unit	*X*_*mean*_	*S*_*x*_	*C*_*v*_	*C*_*x*_	*X*_*min*_	*X*_*max*_
Bayan Lepas	*T*_*max*_	°C	31.89	0.75	2.36	0.52	30.15	34.78
	*T*_*min*_	°C	24.94	0.51	2.07	0.47	23.73	26.90
	*RH*	%	78.74	4.16	5.29	− 1.05	64.66	85.92
	*S*_*w*_	m/s	1.93	0.34	17.99	0.86	1.20	3.25
	*R*_*s*_	MJ m^−2^	18.32	2.14	11.69	1.06	12.72	28.49
	*E*_*p*_	mm	3.89	0.66	17.11	1.17	2.70	6.27
Ipoh	*T*_*max*_	°C	32.99	0.84	2.55	0.35	30.59	35.80
	*T*_*min*_	°C	23.93	0.53	2.24	− 0.03	22.40	25.40
	*RH*	%	80.54	3.92	4.87	− 0.53	68.33	88.71
	*S*_*w*_	m/s	1.52	0.32	21.18	− 1.28	0.62	2.15
	*R*_*s*_	MJ m^−2^	17.79	1.49	8.40	− 0.19	13.31	21.72
	*E*_*p*_	mm	4.29	0.49	11.47	0.007	3.21	5.69
KLIA Sepang	*T*_*max*_	°C	32.20	0.81	2.54	0.63	30.29	34.77
	*T*_*min*_	°C	24.42	0.49	2.02	0.10	23.24	25.69
	*RH*	%	79.62	4.13	5.19	− 0.82	63.51	87.51
	*S*_*w*_	m/s	1.87	0.27	14.62	0.45	1.15	2.81
	*R*_*s*_	MJ m^−2^	17.55	2.38	13.59	0.59	11.12	24.76
	*E*_*p*_	mm	4.17	0.48	11.66	1.13	3.20	6.12
Kuantan	*T*_*max*_	°C	32.17	1.24	3.88	− 0.63	28.52	34.89
	*T*_*min*_	°C	23.71	0.64	2.70	− 0.64	21.14	25.53
	*RH*	%	84.29	3.01	3.58	0.33	77.33	92.39
	*S*_*w*_	m/s	1.64	0.30	18.49	0.64	0.91	2.65
	*R*_*s*_	MJ m^−2^	17.26	2.16	12.56	− 0.47	11.73	22.39
	*E*_*p*_	mm	3.79	0.53	13.64	− 0.26	2.69	5.13

In the table, the *X*_*min*_*, X*_*max*_, *X*_*mean*_*, S*_*x*_*, C*_*x*_*, and C*_*v*_ represent the minimum, maximum, mean, standard deviation, skewness, and coefficient of variation of the weather parameters, respectively. It is apparent from this table data that the Ep minimum value was measured at Kuantan station, whereas the maximum value was recorded in the Bayan Lepas station. This might be due to the rate of relative humidity, which is inversely associated to evaporation; Kuantan station has the highest relative humidity rate, and Bayan Lepas station represents the lowest rate. Conversely, the coefficient of variation and maximum skewness of Ep were also measured in the Bayan Lepas station, while the minimum value was recorded in Ipoh. A positive value of skewness implies that the information is not symmetric and does not adhere to the normal distribution.

### Partitioning of data and input selection

Selecting the suitable predictors is one of the most crucial steps in developing a robust predictive model^52^; different input combinations of meteorological parameters were examined in this study to successfully plot input–output model and improve the predictive ability of ML models. This will enable a better practical comprehension of how every input parameter affects the evaporation estimate in that region^53^. There are certain conscious choices for choosing these combinations. First, for the purpose of comparison, input variables to the models of machine learning (RF, DNN, and CNN) were chosen according to the required meteorological aspects in the two proposed empirical models (Thornthwaite and Stephens & Stewart). Second, the input variables (predictors) were chosen with reference to the PCC ^54^. The Pearson correlation method is the test statistics that quantifies the statistical correlation, or association, among two continuous parameters. It is identified as the best technique of measuring the correlation between parameters of interest since it is based on the covariance method ^55^. It gives data about the association or correlation magnitude and the direction of the correlation. The two parameters can be negatively or positively associated and there is no relationship among the two parameters if the PCC is 0. To show the applicable features of the environmental parameters to estimate monthly evaporation, the PCC interpretations and ranges are used as displayed in Table 2. The PCC were employed to find the meteorological parameters showing the greatest effect on the estimates of evaporation, and the results are shown in Table 3.

**Table 2:** Ranges and analysis of the Pearson correlation coefficient (PCC).

PCC ranges	Analysis
0.00 < 0.09	Insignificant
0.10 < 0.19	Weak
0.20 < 0.39	Moderate
0.40 < 0.59	Moderately strong
0.60 < 0.79	Strong
0.80 < 1.00	Extremely strong

**Table 3:**
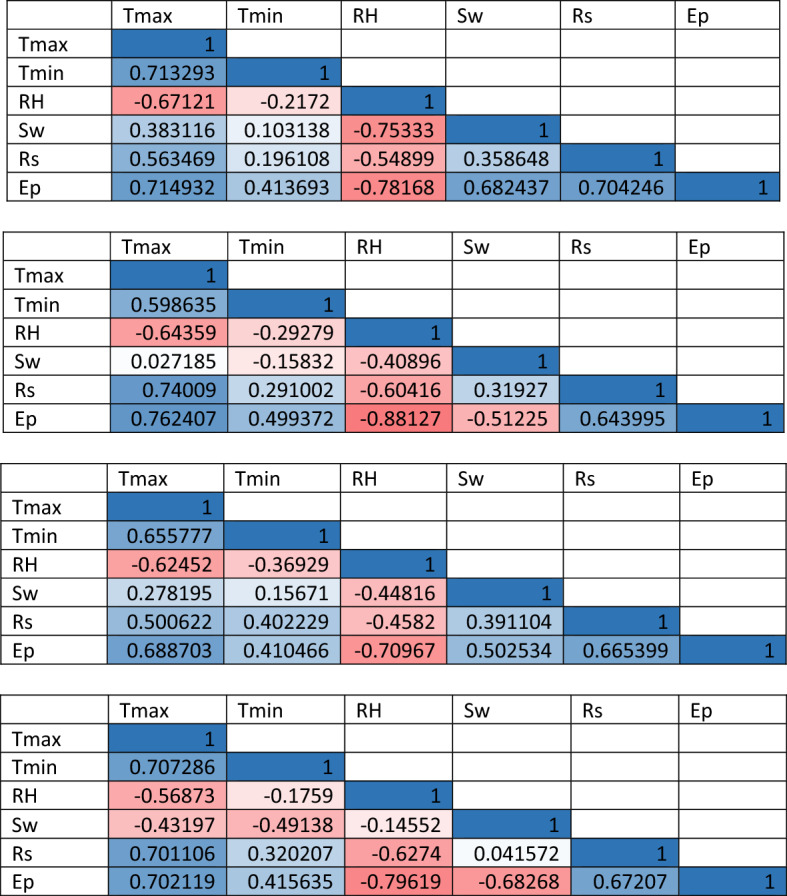
Pearson correlation coefficient values between the meteorological variables measured at Bayan Lepas, Ipoh, KLIA Sepang, and Kuantan stations.

The outcomes are listed in Table 3, indicating that the T_max_, T_min_, RH, S_w_, R_s_ were all related to a certain extent with Ep and therefore can play a crucial role in predicting the evaporation parameter for the data gathered at all stations. Particularly, at all stations, the T_max_ and RH parameters have the strongest relationship with Ep. Thus, the T_max_ and RH will be employed in all input combinations in order to increase the Ep estimation accuracy. Earlier studies also suggested that T_max_, T_min_, RH, S_w_, and R_s_ are some of the most significant predictors of evaporation ^56,57^.

**Figure 3:**
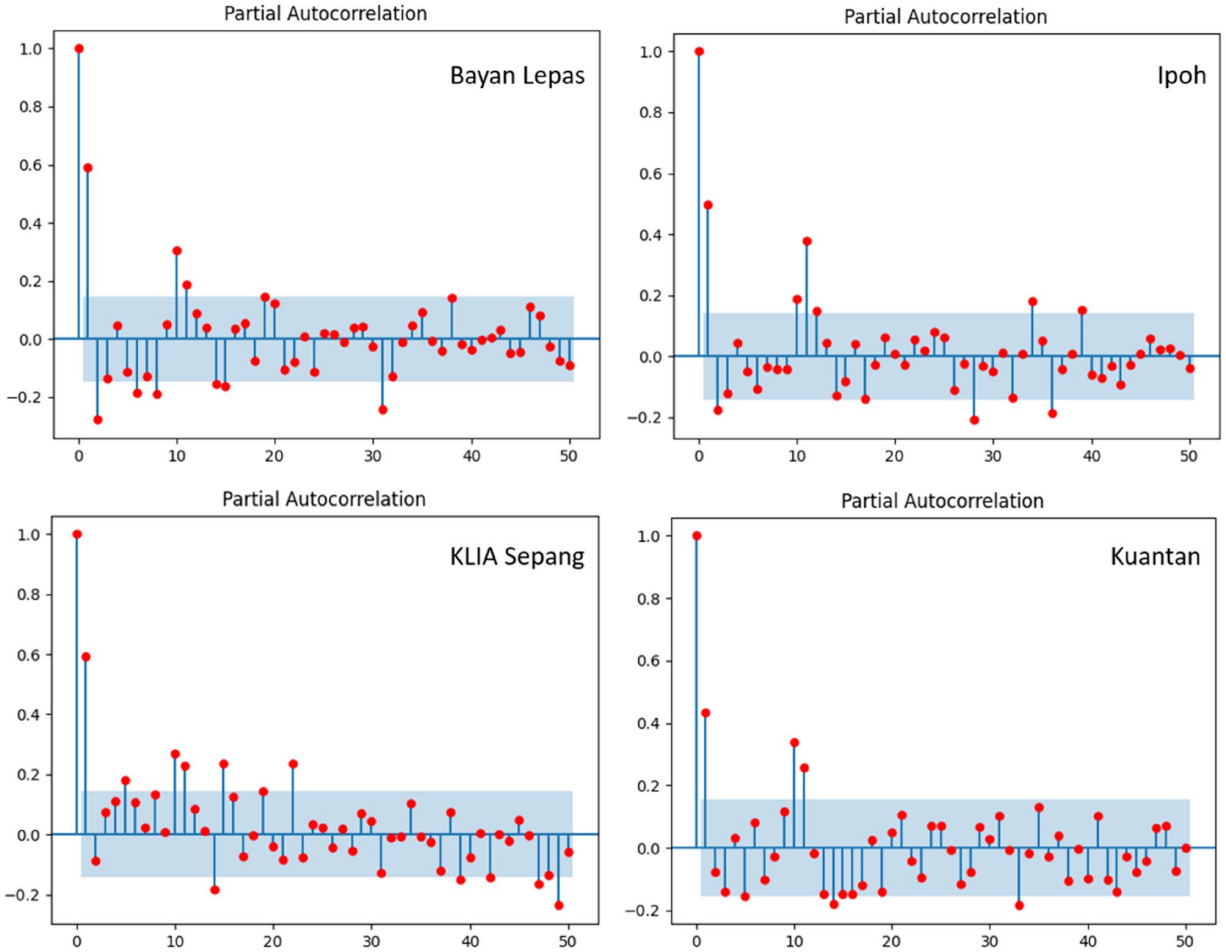
Partial autocorrelation for Bayan Lepas, Ipoh, KLIA Sepang and Kuantan stations (Monthly).

The current research has also evaluated the effects of the input parameter Ep in improving the prediction accuracy for evaporation. In this regard, the records of input data were chosen with reference to how the previous records were associated with the estimated output value. As illustrated in Fig. 3, at each of the stations, the autocorrelation examination for the recorded time series on monthly basis for the Ep rate showed that the correlation declined significantly once it went beyond the previous second lag-time record. This shows that the previous record of second evaporation rate affected the evaporation rate at any time. Therefore, based on the past pan evaporation rate records with the advantage of the correlation analysis, the highest lag times of two previous records were employed as the model input when building the proposed models on monthly basis.

Accordingly, in the current study, nine different input scenarios were considered for the models (Table 4). Each climatic data set was divided into two sets, in which 80% was employed for model calibration (training) while 20% was used for validation (testing). Thus, the dataset was partitioned by taking the initial years for training and the remaining years for testing. However, the evaluation of ML approaches is extremely sensitive to the adopted data partitioning scheme. Therefore, the k-fold CV technique would be used. Despite the high computational cost associated in the CV method, it is regarded as one of the reliable prevention methods against overfitting^58^. The current study intends to perform a comprehensive assessment for testing AI ability and using practical models for predicting Ep levels on a monthly basis in the Bayan Lepas, Ipoh, KLIA Sepang, and Kuantan regions.

**Table 4:** Input combinations of meteorological variables used for ML models.

No.	Model			Input combinations
	RF	DNN	CNN	
1	RF-1	DNN-1	CNN-1	T_a_
2	RF-2	DNN-2	CNN-2	T_a,_ R_S_
3	RF-3	DNN-3	CNN-3	RH
4	RF-4	DNN-4	CNN-4	RH, T_max_
5	RF-5	DNN-5	CNN-5	RH, T_max_, T_min_
6	RF-6	DNN-6	CNN-6	RH, T_max_, T_min_, S_w_
7	RF-7	DNN-7	CNN-7	RH, T_max_, T_min_, R_s_
8	RF-8	DNN-8	CNN-8	RH, T_max_, T_min_, R_s_, S_w_
9	RF-9	DNN-9	CNN-9	RH, T_max_, T_min_, R_s_, S_w_, Ep

## Methodology

### Empirical models used for monthly Ep prediction

In this research, Stephens & Stewart and Thornthwaite were selected for comparison as the two empirical techniques, as they are regarded to be widely employed methods^59^, taking into account the number of meteorological inputs required as well as the availability of the data.

#### Stephens & Stewart

This technique is also commonly referred as the ‘Fractional Evaporation-Equivalent of Solar Energy’ approach by Stephens & Stewart^60^. As presented in Eq. (1), Stephens & Stewart suggested that by employing measured radiation Qs, better results were achieved when there is availability of data and it also allows correlating with temperature:

1 $$Ep= \left(0.0082Ta-0.19\right)\left(\frac{Qs}{1500}\right) \times 25.4,$$

where $Ta$, $Ep$, and $Qs$ represent mean air temperature (Fahrenheit), evaporation (mm), and solar radiation (cal cm^−2^ day^−1^). Stephens & Stewart also recommended carrying out additional research in other regions to set such relationships under different weather conditions.

#### Thornthwaite

Thornthwaite^61^ employed practical data to identify the relationship that exists between mean monthly temperature (Ta) and probable evaporation (Ep), and then set standardisation to a 30-day month with 12 h of sunlight each day. The potential evaporation (Ep) is calculated by employing Thornthwaite technique; the following expression is employed to calculate the Monthly Thornthwaite Heat Index ($I$):

2 $$i={\left(\frac{Ta}{5}\right)}^{1.514},$$

where $Ta$ represent mean monthly temperature (°C).

The Annual heat index $\left(I\right)$ is calculated as the sum of the Monthly Heat Indices $\left(i\right)$:

3 $$I=\sum_{i=1}^{12} i.$$

The potential evaporation $Ep$ for each month is calculated using the following equation:

4 $$Ep=16 \cdot {\left(\frac{10 \cdot Ta}{I}\right)}^{a},$$

where $$a$$ is:

5 $$a=\left(675 \times {10}^{-9} \times {I}^{3}\right)-\left(771 \times {10}^{-7}\times {I}^{3}\right)-\left(1792\times {10}^{-5}\times I\right)+0.49239.$$

$Ep$ for a given month is given by the expression:

6 $$Ep={Ep}_{Obtained} \cdot \frac{N}{12} \cdot \frac{d}{30}\,\, \left(\mathrm{mm}\right).$$

N and d denote the number of theoretical monthly sunshine hours and days in the month, respectively.

### ML models used for monthly Ep prediction

Three ML frameworks were included in the current study to estimate evaporation, i.e., RF, DNN, and CNN. The TensorFlow framework geared with an NVIDIA GeForce GTX 1080 Ti GPU was employed to conduct training and testing of the machine learning models.

#### Random Forest (RF)

**Figure 4:**
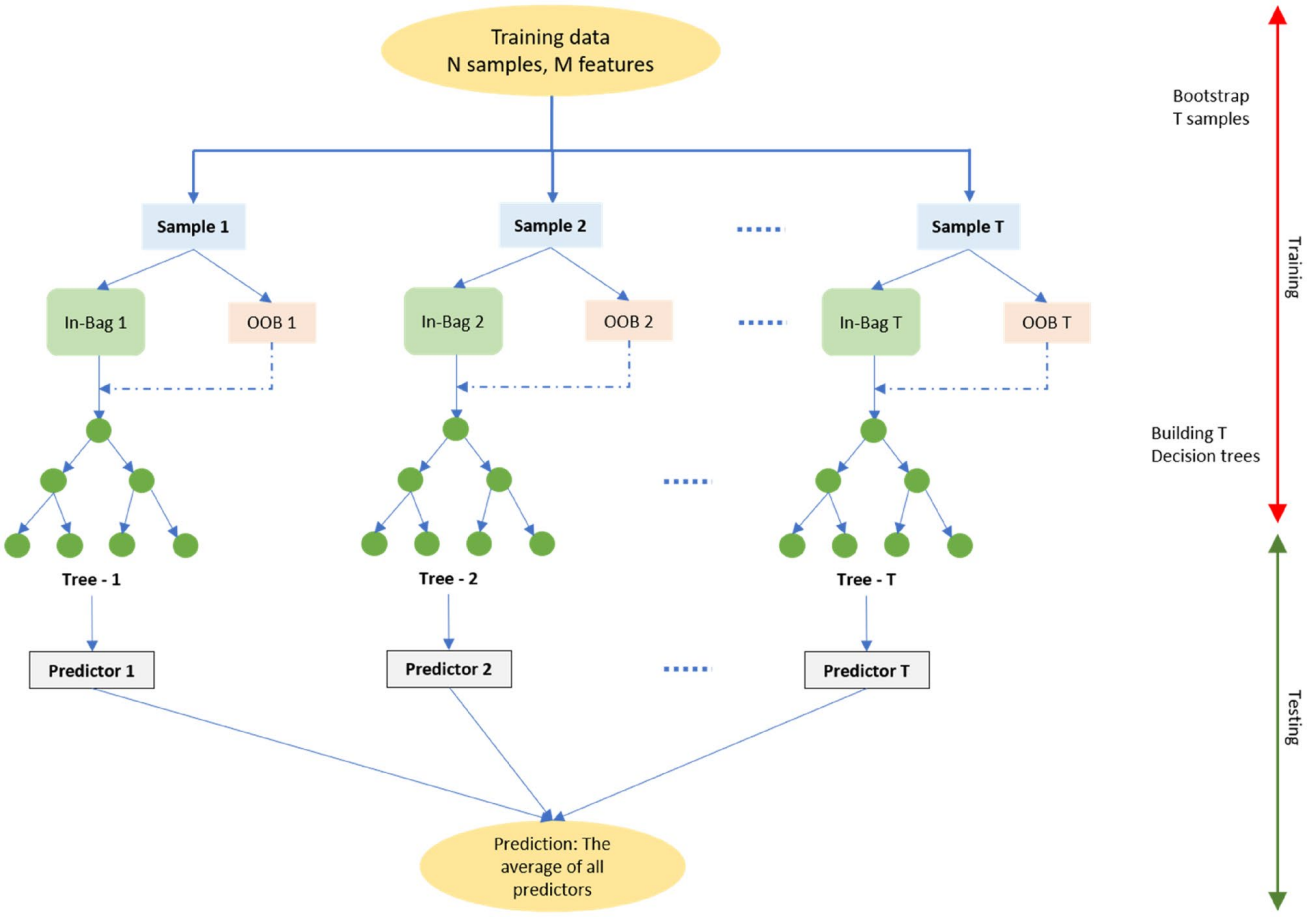
General architecture of the RFs model.

The Random Forest algorithm is an effective tree-based ensemble learning algorithm, which is known for its excellent performance. It has a broad range of applications, including regression, classification as well as unsupervised learning^62^. The RFs model was put forward by Breiman^63^, which employed Breiman’s ‘bagging’ idea to ensemble a set of decision trees that possess controlled variation. The data set excluded in the development of the model signified as out-of-bag (OOB) samples is used to assess the general problems (Fig. 4). This also offers a quantitative measurement pertaining to contribution of each input auxiliary data towards the prediction step, referred as RF variable importance^64^. The functioning of Random Forest algorithm in general follows these steps: (i) collect and then re-sample the original training data several times; (ii) select a random set of features for every re-sampling step; (iii) estimate a decision tree based on a re-sample and a random set of features; (iv) to obtain a single decision tree, a set of estimated decision trees is gathered. It can be noted that RF is rather insensitive towards noise as well as overtraining, It has been broadly employed to solve complicated as well as non-linear hydrological engineering issues ^65,66^. Additional details about the random forest model theories can be noted in ^63^.

In this study, different hyperparameters were employed in RF in order to determine the best ones that can achieve the highest accuracy with regards to prediction, such as:

1. *The total number of trees needed to generate the forest (Ntree)* This parameter is regarded to be a determinant factor when it comes to conducting predictions with RF.
2. *The tree’s maximum depth* With regards to Random Forest, the maximum depth of a tree refers to the longest path between the leaf node and the root node.
3. To identify the best split, the following features need to be kept in mind:
  max_features {“auto”, “sqrt”, “log2”}.
  If “auto”, then max_features = n_features.
  If “sqrt”, then max_features = sqrt (n_features).
  If “log2”, then max_features = log2 (n_features).

#### Deep neural network (DNN)

**Figure 5:**
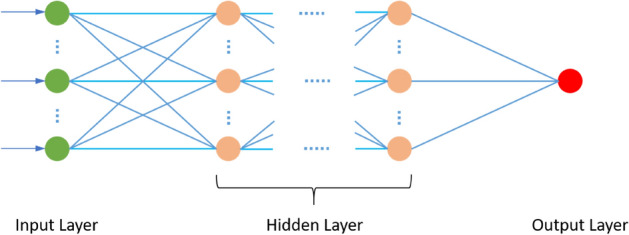
The basic structure of DNN.

In the deep learning field, DNN are regarded to be a key technique^42^. The fundamental framework has been built by considering the brain’s functioning and biological structure to enable machines to achieve intelligence that is more human-like. The basic version pertaining to DNN represents a hierarchical collection of neurons that transmit messages to other neurons as per the input, thus resulting in the development of a complex network learning based on the feedback mechanism. Figure 5 shows the typical structure pertaining to DNN, which includes one input layer, one output layer and numerous hidden layers. As shown in Fig. 5, the balls denote the neurons, wherein each link that exists between neurons is represented by a cause-effect chain that can be trained and learned. The layers remain fully connected, in which any particular neuron in one-layer stays connected to each of the neuron in the next layer. The entire DNN model is made up of a linear function outlined in Eq. (7) as well as an activation function as shown below:

7 $$a= \sum {w}_{i}{x}_{i}+ {b}_{i},$$

where ${x}_{i}$ represents the input value pertaining to each neuron; ${w}_{i}$ denotes the coefficient pertaining to linear relationship and ${b}_{i}$ defines the bias. Presuming there are L hidden layers with regards to the DNN, the output value calculation can be represented as follows:

8 $$f\left(x\right)=f\left[{a}^{L+1} ({h}^{L}\left({a}^{L} \left( \ldots \left({h}^{2}\left({a}^{2}\left({h}^{1}\left({a}^{1} \left(x\right) \right) \right) \right) \right) \right) \right)\right] {a}^{L}\left(x\right)= {W}^{L}+b,$$

where $L$ denotes the $Lth$ layer; $x$ signifies the matrix of input variables; $b$ and $W$ indicate high dimensional matrix and $f(x)$ indicates the introduced activation function to boost the nonlinearity pertaining to the neural network in order to approximate any nonlinear function with regards to numerous nonlinear models. Amongst all of these activation functions, the rectified linear unit (ReLU) activation function, i.e. ReLU($x$) = max($x$, 0), has now become the most popular activation functions employed in the deep learning literature as well as applications ^67^.

Determination of the values of $W$ and $b$ is determined automatically by taking into account the minimum value pertaining to the loss function in the training process. The difference that exists between the actual and predicted values is determined by employing the loss function. The model’s robustness gets better when there is a smaller value of loss function. Finally, the output layer is regarded to be the final layer of the network. In this research work, testing of different hyperparameters is done to choose the best architecture that can offer the highest evaluation metrics that will help determine the DNN's optimal structure. The hyperparameters include: (1) The total number of fully connected layers, (2) kinds of activation functions that exist amongst layers, (3) percentage of dropout as well as number of dropout layers, (4) loss function, (5) batch size, (6) optimiser, (7) number of epochs and (8) Learning rate. The put forward DNN model’s best architecture with regards to prediction of evaporation includes the following layers:

1. Fully connected layers with 64 nodes and ReLU activation function.
2. Dropout with 0.1%.
3. Fully connected layers with 128 nodes and ReLU activation function.
4. Dropout with 0.1%.
5. Fully connected layers with 1 node and Linear activation function.

The final hyperparameters are:

1. The learning rate: 0.001.
2. Loss Function: Mean Square Error (MSE).
3. Optimizer: ADAM.
4. Epochs: 500.
5. Batch size: 8.

#### Convolutional neural network (CNN)

CNN is a renowned and extensively utilised deep learning structure. First recommended by LeCun et al. ^68^, CNNs are still a broadly deployed model for image processing and examination due to their capability to mine and decompose features and secure spatial correlations between data in one or two dimensions^69^. Convolutional neural network usually pertains to a 2-dimensional CNN, which is typically utilised for image classification. There are other kinds of CNNs like 1-dimensional (1D-CNN) and 3-dimensional (3D-CNN) which are also utilised in real-life engineering applications. Notably, all CNNs possess the same attributes and follow the same methodology. However, the key dissimilarity is the input data dimensionality and the way the filter (feature detector) moves over the data. In this work, we utilised 1D-CNN for pan evaporation estimation because of its advanced performance and minimal computational intricacy. CNNs comprises two key parts^70^: the first comprises convolutional filtering for mining attributes hierarchically and the second is a fully-connected layer for computing the output value from manifold input values comprising fully-connected neuron layers. The fully connected layers are quite similar to the multilayer perceptron (MLP) layers. The MLP is a feed forward neural network which utilises stochastic gradient descent backpropagation algorithmic for network optimisation. In fact, ordinary artificial neural networks (ANNs) solely comprise the second part; thus, the feature extraction stage is the key difference between CNNs and normal ANNs.

**Figure 6:**
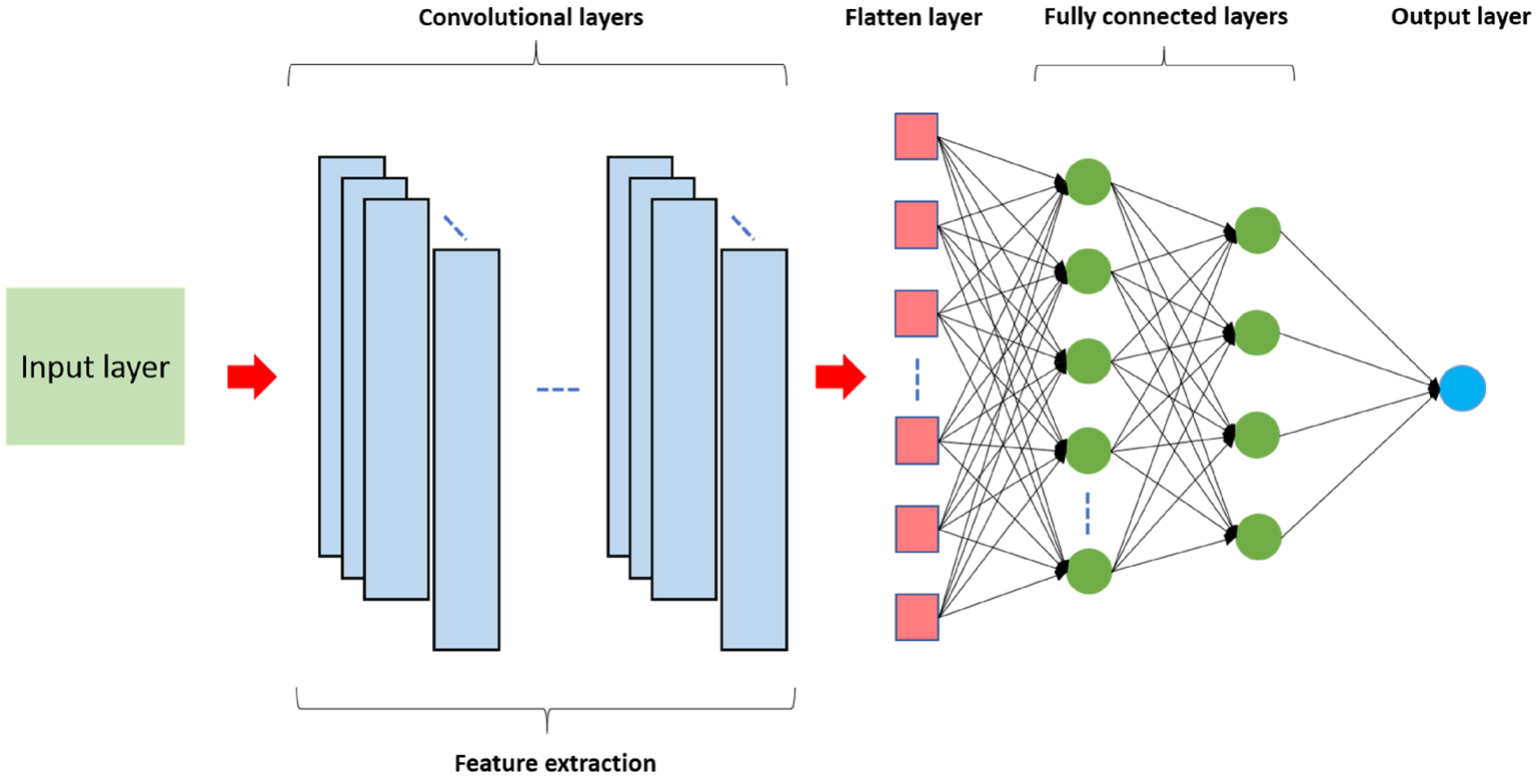
General architecture of the CNN model.

The CNN design generally encompasses an input layer, an output layer and few random numbers of hidden layers among them. A typical CNN setup is depicted in Fig. 6. The input layer is responsible for receiving the signal (input data) as well as transmitting it to the hidden layer(s). Hidden layers can be defined as the computational engine pertaining to the model. These could include one or more dropout layer, convolutional 1D layer, max-pooling layer as well as a flatten layer based on the problem. The CNN’s chief building block is the convolutional layer that includes one-dimensional filters/kernels that enable extracting the features via the input signal, an activation function for establishing neurons' threshold limit and kernel size to denote the filter length. There are many commonly utilised activations functions like the ReLU, tanH, Softmax, and Sigmoid. Each of these have a particular use. The hidden information in the input data can be identified and excerpted via convolutional filters. Towards the end of the convolution layers, the learning features are generally flattened to a single long vector array and tend to pass via fully connected layers prior to employing the output layer for prediction. The flatten layer transforms the convolutional/pooling/dropout layers’ output to one dimension and then transmits the data to the output layer. To the neurons in the network, the dropout layer (should it be employed) randomly assigns zero weights, making it less sensitive to minor variation, thereby enhancing the model’s accuracy regarding unseen data. The 1D-CNN’s last layer would be the output layer that contains one neuron for yielding the desired output. To summarise, there exist three kinds of layers which constitute the CNN: the convolutional layers, fully connected (FC) layers, and pooling layers. Once these layers are arranged, a CNN architecture would be created.

In this work, many meteorological variables, such as T_max_, T_min_, RH, S_w_, and R_s_ were applied to CNN to estimate the pan evaporation rate. Iterative parameter tuning helped CNN fit the dataset. To determine the precise CNN structure, several hyperparameters were evaluated to determine the optimal structure to offer the most precise assessment metrics. These hyperparameters comprise convolutional layer count, layer-specific feature map count, filter size, pooling layer category, activation function categories between layers, dropout percentage and numbers, fully-connected layer count, loss function, learning rate, epoch count, batch size, and optimiser. Typically, CNN is built using dense and convolutional layers. Pooling layers might be included in such networks; the layers are inserted between convolutional layers to decrease problem dimensions and identify critical features. Nevertheless, this study does not consider pooling layers because excess parameter count is tolerable for time series forecasts, and recent studies are critical about the need for pooling layers^71^. Moreover, researchers assert that adequately sized convolutional layers suffice for networking function without adding additional layers^71^. The sequential model is typically used for Python programming, and it was used for this step too. It provides a straightforward technique to create a CNN structure using Keras since it facilitates building the structure based on layers. During CNN training, the objective is to optimise the loss function representing the objective in the neural network structure. The function is based on MSE. This study also employed the dropout technique to reduce overfitting. Dropout is a widely-used regularisation technique (creative a more representative CNN weight range by creating a new scale), and the values were 0.1 and 0.2. A batch size of 16 and 500 epochs were chosen for training the model based on the above architectural configuration and several trials. Adam algorithm^72^ was employed to adjust network weights to reduce loss function and determine network performance with a learning rate of 0.001 and momentum rate of 0.7.

The one-dimensional CNN structure proposed in this study comprises the following layers for optimal performance concerning evaporation prediction:

1. CNN with one convolutional layer and 32 filter with kernel_size = 2 and activation = ‘relu’.
2. Dropout with 0.2%.
3. Flatten layer (used as a connection between Convolution and the Dense layers).
4. Fully connected layers with 128 nodes and ReLU activation function.
5. Dropout with 0.1%.
6. Fully connected layers with 256 nodes and ReLU activation function.
7. Dropout with 0.1%.
8. Fully connected layers with 1 node and Linear activation function.

The final hyperparameters are:

1. Learning rate: 0.001.
2. Loss function: MSE.
3. Optimizer: ADAM.
4. Epochs: 500.
5. Batch size: 16.

### Performance evaluation

Choosing the appropriate performance indicators is crucial since every indicator has its own properties. In addition, knowing the strengths of each statistical measure can provide a better understanding of how the model perform. Therefore, in this study, model predictive performance was evaluated by utilising numerous well-known statistical indicators. These indicators are defined below:

1. *R*^*2*^ the coefficient of determination informs the correlation between the real and estimated outputs; it has a value range of 0–1 (both limits included). Zero indicates a random framework, while one represents optimal fit. R^2^ is very popular and makes comparing models easier and more consistent. It attempts to measure how well a regression model is fit a dataset, providing evaluators with an instant understanding of the model’s performance.
  9 $${R}^{2}= \frac{\sum_{i=1}^{n}\left(y- \overline{y }\right) (\widehat{y}- \overline{\widehat{y} })}{\sqrt{\sum_{i=1}^{n}{(y- \overline{y })}^{2 } } \sum_{i=1}^{n}{(\widehat{y}- \overline{\widehat{y} })}^{2} }.$$
2. *MAE* the absolute difference between the actual and predicted output. High errors caused by outliers are not penalised by MAE. Furthermore, it provides a consistent indicator of how precise the model performs.
  10 $$MAE= \frac{1}{n}\sum_{i=1}^{n}\left|y- \widehat{y}\right|.$$
3. *MSE* the average squared difference between predicted and actual output. By squaring the errors, the MSE penalises the model for having large errors. Furthermore, for minor errors, it efficiently converges to the minima.
  11 $$MSE= \frac{1}{n} {\sum_{i=1}^{n}{(y- \widehat{y})}^{2}}.$$
4. *RMSE* it is the square root of the average value of error squares concerning the real and estimated values. In assessing the performance of a regression model, RMSE is more commonly used than MSE. In addition, RMSE is straightforward and easily distinguishable. RMSE has the added benefit of penalising large errors, making it more acceptable.
  12 $$RMSE=\sqrt{\frac{{\sum_{i=1}^{n}(y- \widehat{y})}^{2}}{n}}.$$
5. *RAE* the difference between real and forecasted values are gathered and normalised. RAE is reliable in some cases because it protects against outliers.
  13 $$RAE= \frac{\sum_{i=1}^{n}\left|y- \widehat{y}\right|}{\sum_{i=1}^{n}\left|y- \overline{y }\right|}.$$
6. *NSE* it represents a normalised metric determining the relative intensively of residual variance (noise) when determined against the calculated variance (information). The NSE is still widely used in hydrologic modelling, in part since it normalises performance of the model into an understandable scale.
  14 $$NSE=1- \frac{\sum_{i=1}^{n}{\left(y- \widehat{y}\right)}^{2}}{\sum_{i=1}^{n}{\left(y- \overline{y }\right)}^{2}},$$
  where n is sample count, y denotes the true output, $\widehat{y}$ denotes the predicted values, and $\overline{y }$ is the true output average.
7. *Taylor diagram (TD)* Besides the above-mentioned statistical factors, Taylor diagram^73^ was also used to calculate the accuracy of the modelling methods taken into consideration and their extent of similarity. The diagram is normally used in climate-based studies^74^. These diagrams can underline the accuracy of models’ estimates by comparing the predicted and measured values by visualising a series of elements on a polar plot. The diagram’s azimuth angle illustrates the correlation coefficient between the predicted and measured values, whereas the standard deviation value of the modelled data from observations is shown by the radial distance from the origin.

**Figure 7:**
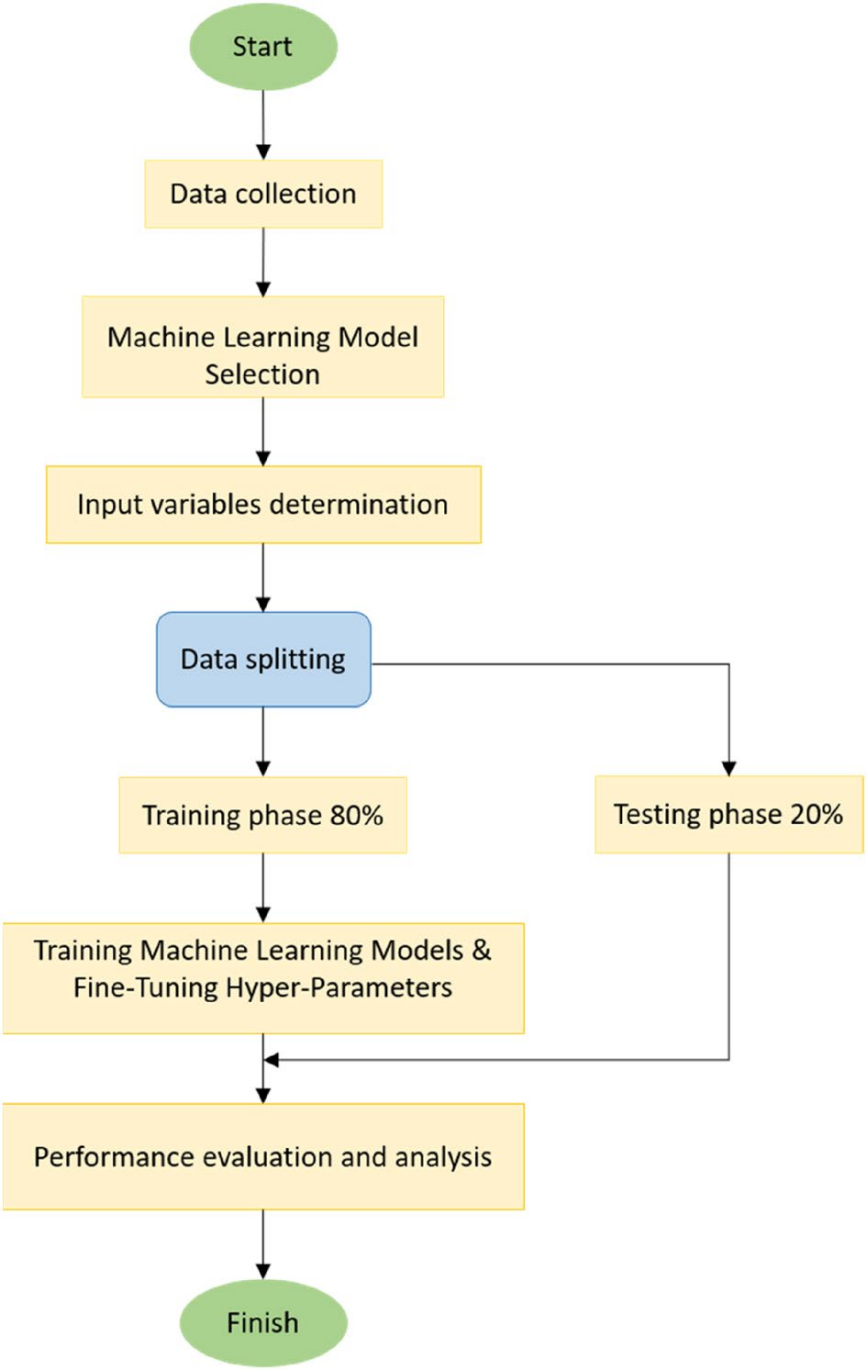
The process of developing a prediction model.

As a conclusion to the performance and training evaluation procedures for the ML models that are proposed, a flow chart is devised which is displayed in Fig. 7. The detailed procedure employed in this approach has been illustrated in the flow chart.

## Results and discussion

### Estimation of monthly Ep using empirical models

**Figure 8:**
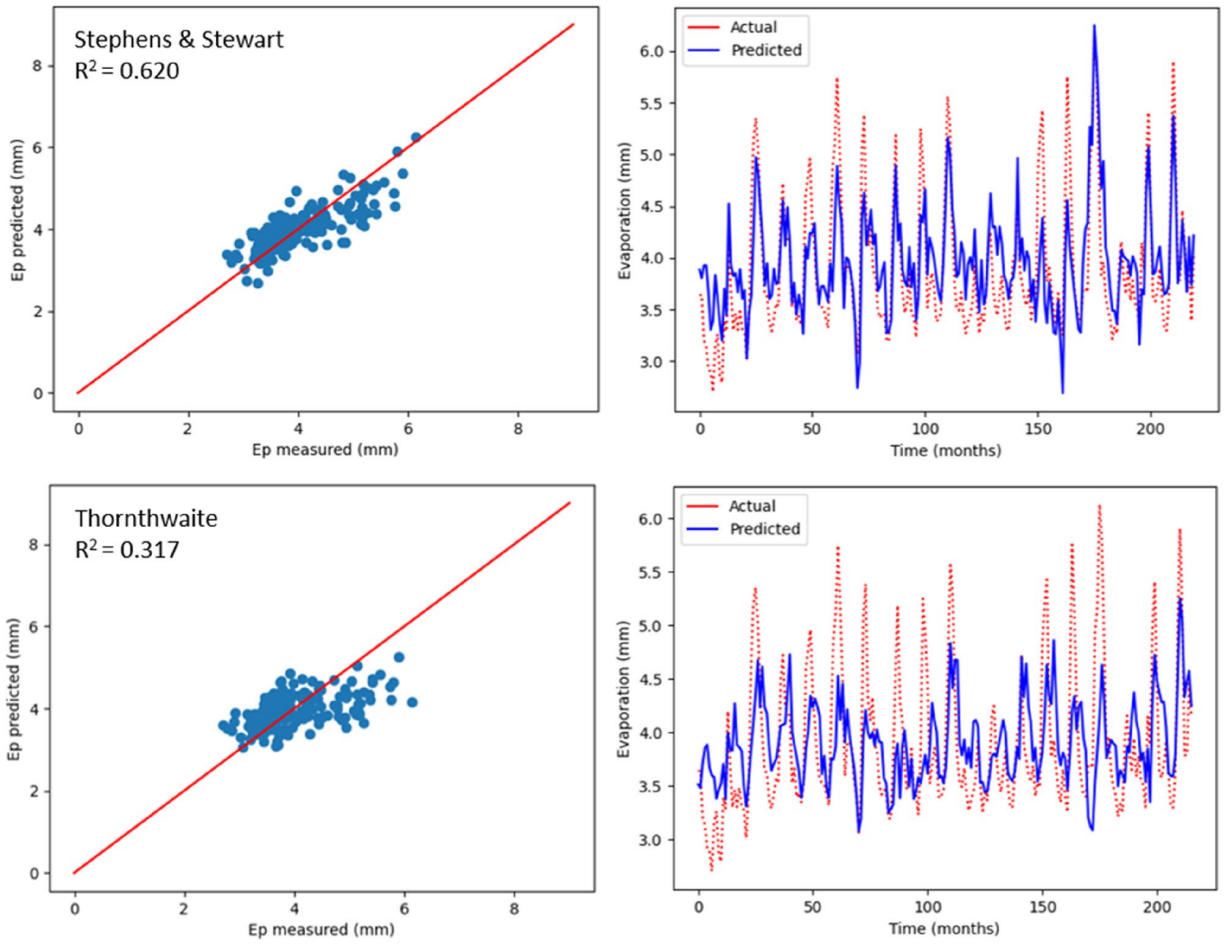
Scatter plot of measured Ep versus predicted Ep for the proposed empirical modles for Bayan Lepas station.

**Figure 9:**
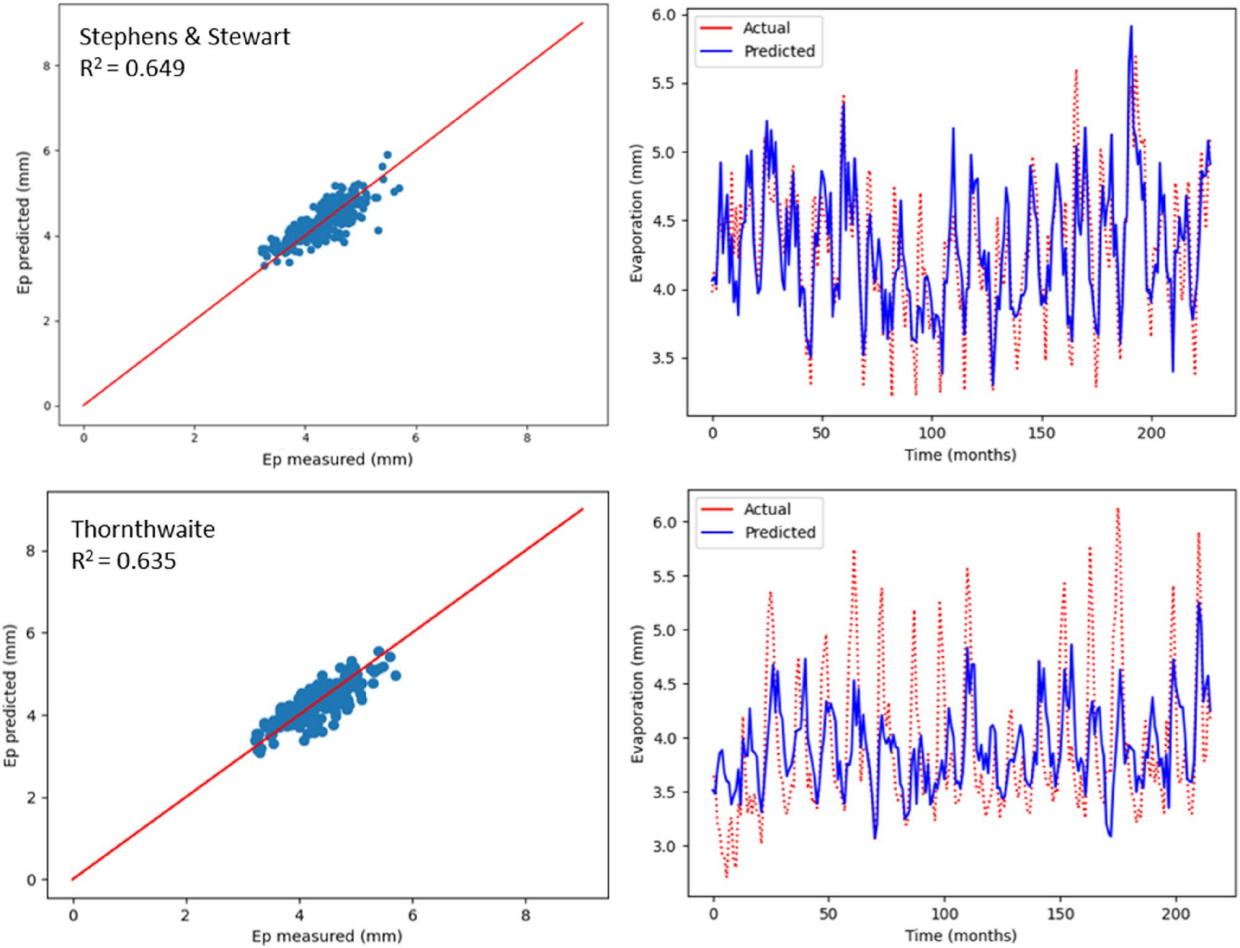
Scatter plot of measured Ep versus predicted Ep for the proposed empirical modles for Ipoh station.

**Figure 10:**
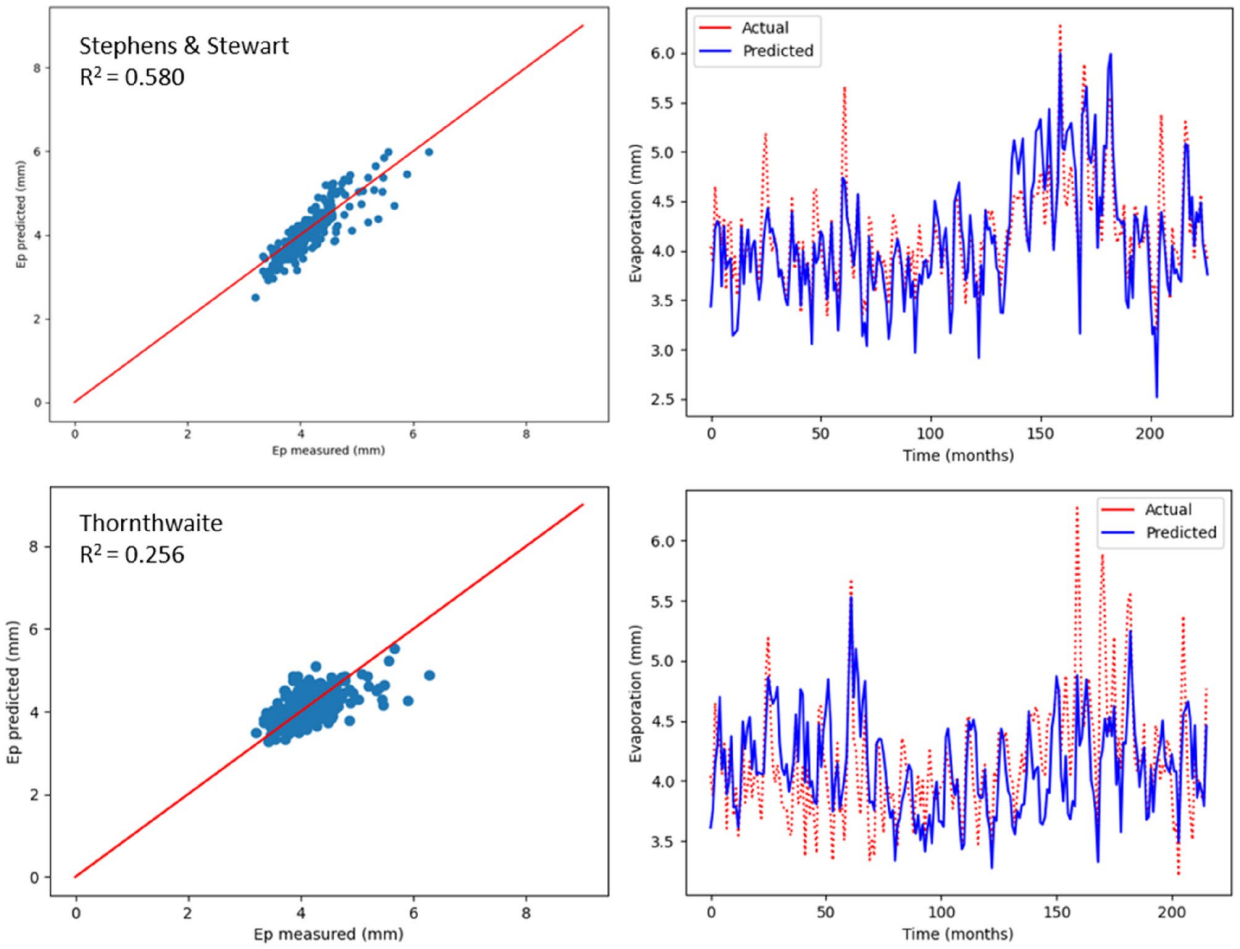
Scatter plot of measured Ep versus predicted Ep for the proposed empirical modles for KLIA Sepang station.

**Figure 11:**
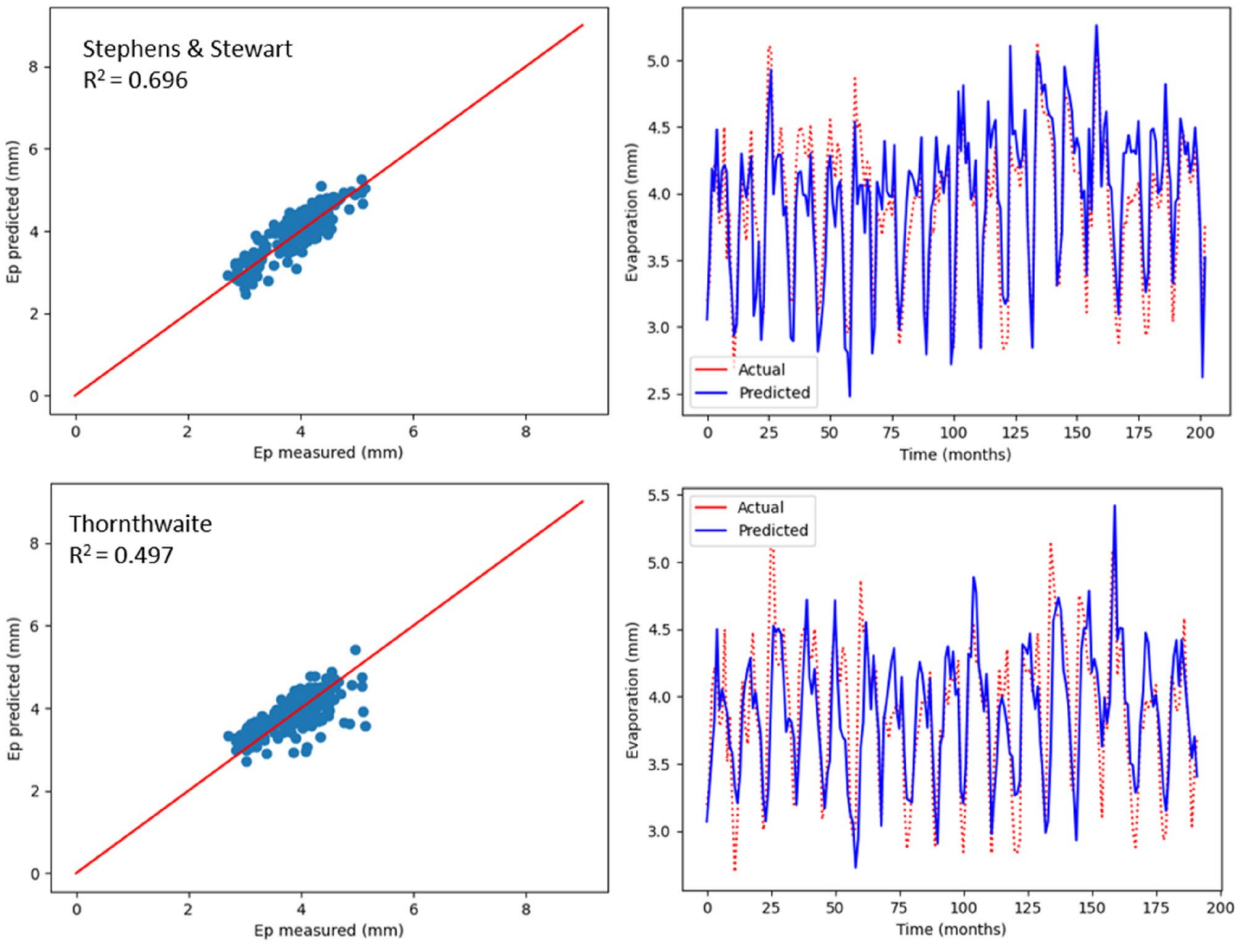
Scatter plot of measured Ep versus predicted Ep for the proposed empirical modles for Kuantan station.

As previously mentioned, monthly Ep was estimated using two empirical models, which include radiation-based and temperature-based models. The values relating to R^2^, MSE, MAE, NSE, RAE and RMSE are recorded in Table 5, with respect to the two models used to estimate Ep in Bayan Lepas, Ipoh, KLIA Sepang and Kuantan stations. As indicated by the statistical values shown in Table 5, greater prediction accuracy was noticed with the model based on radiation (Stephens & Stewart) in comparison with the temperature-based model. Above all, the highest R^2^ values (0.620, 0.649, 0.580, and 0.696) and the minimum RMSE values (0.409, 0.292, 0.314, and 0.292) were observed in Stephens & Stewart model for all stations. However, in the Thornthwaite model, values of RMSE increased by approximately average 16%, and the corresponding R^2^ reduced by approximately average 33%. The performance values listed in Table 5 clearly suggest that the Stephens & Stewart model surpassed the Thornthwaite model. It could be due to the inclusion of solar radiation, which generally includes an improvement over only the temperature-based estimation^53^. In Figs. 8, 9, 10 and 11, projected values related to monthly Ep with respect to both the empirical models are plotted against the values measured at stations Bayan Lepas, Ipoh, KLIA Sepang and Kuantan, respectively.

**Table 5:** Statistical results of Stephens & Stewart and Thornthwaite empirical models for prediction Ep at Bayan Lepas, Ipoh, KLIA Sepang and Kuantan stations.

Station	Model	R^2^	MAE	MSE	RMSE	RAE	NSE
Bayan Lepas	Stephens & Stewart	0.620	0.328	0.167	0.409	0.631	0.621
	Thornthwaite	0.317	0.431	0.306	0.553	0.820	0.317
Ipoh	Stephens & Stewart	0.649	0.231	0.085	0.292	0.585	0.650
	Thornthwaite	0.635	0.243	0.088	0.296	0.615	0.636
KLIA Sepang	Stephens & Stewart	0.580	0.244	0.098	0.314	0.670	0.581
	Thornthwaite	0.256	0.325	0.175	0.418	0.891	0.257
Kuantan	Stephens & Stewart	0.696	0.245	0.085	0.292	0.572	0.697
	Thornthwaite	0.497	0.284	0.144	0.380	0.657	0.498

### Estimation of monthly Ep using ML models

**Figure 12:**
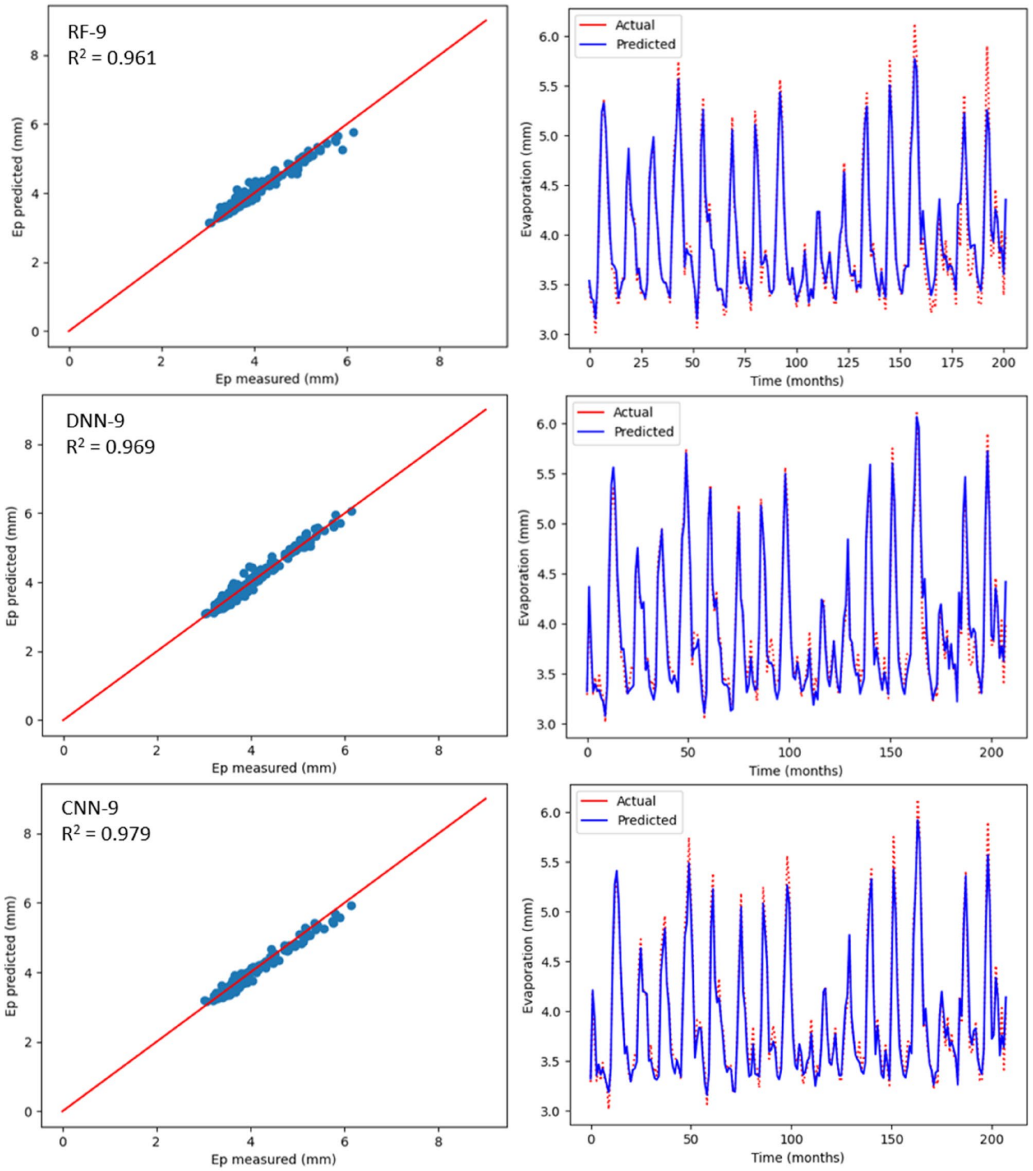
Scatter plot of measured Ep versus predicted Ep for the proposed machine learning models for Bayan Lepas station.

**Figure 13:**
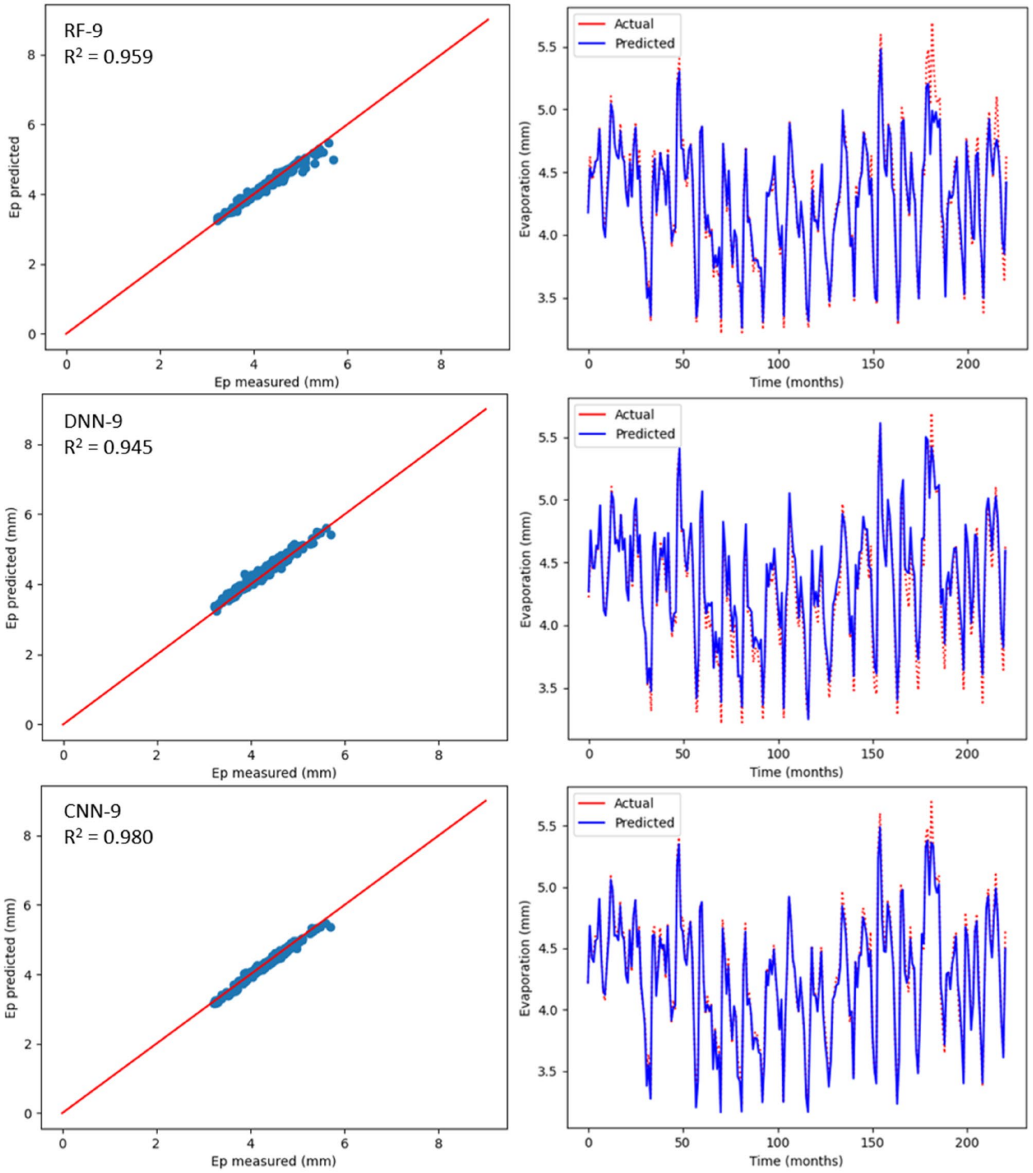
Scatter plot of measured Ep versus predicted Ep for the proposed machine learning models for Ipoh station.

**Figure 14:**
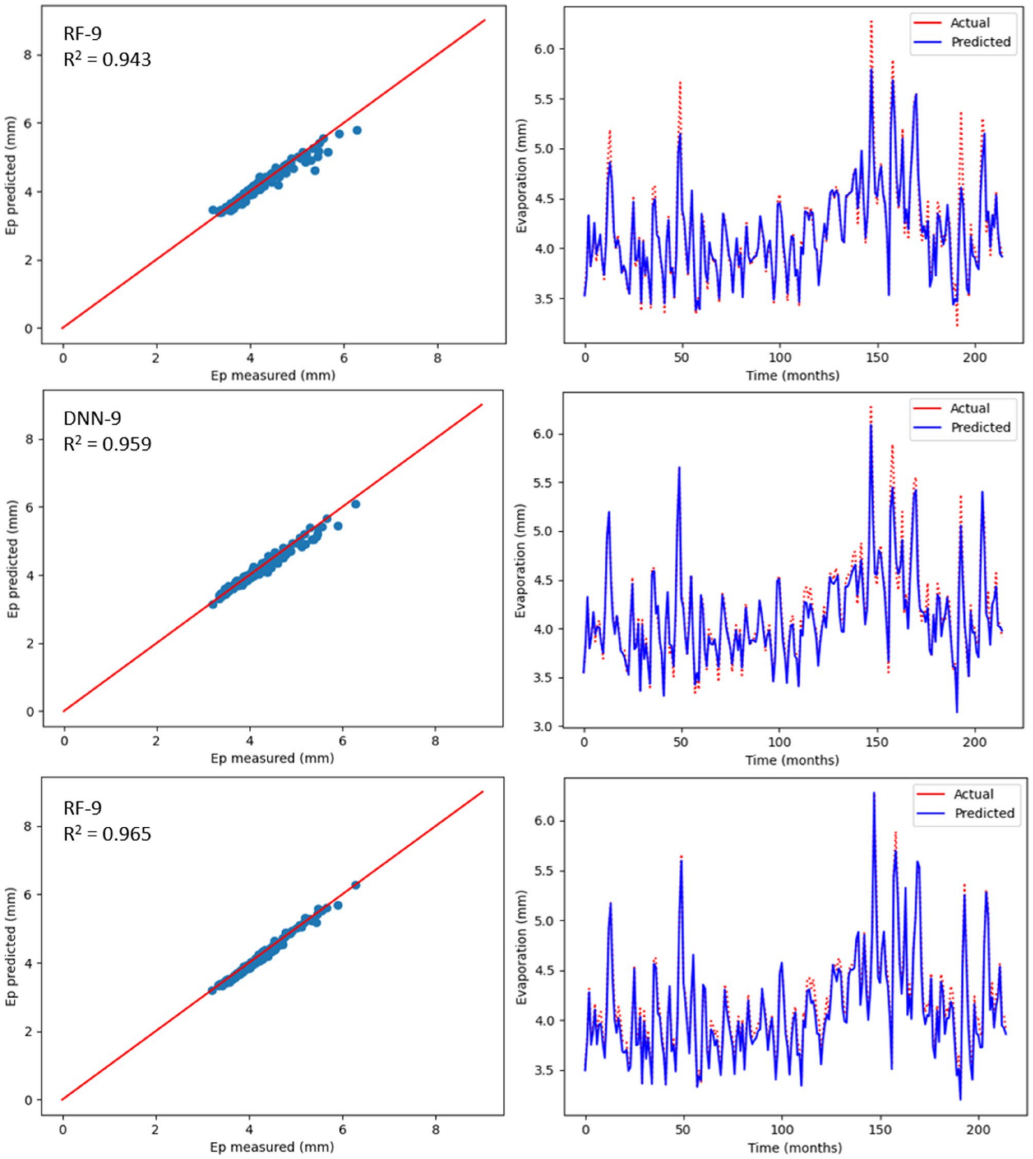
Scatter plot of measured Ep versus predicted Ep for the proposed machine learning models for KLIA Sepang station.

**Figure 15:**
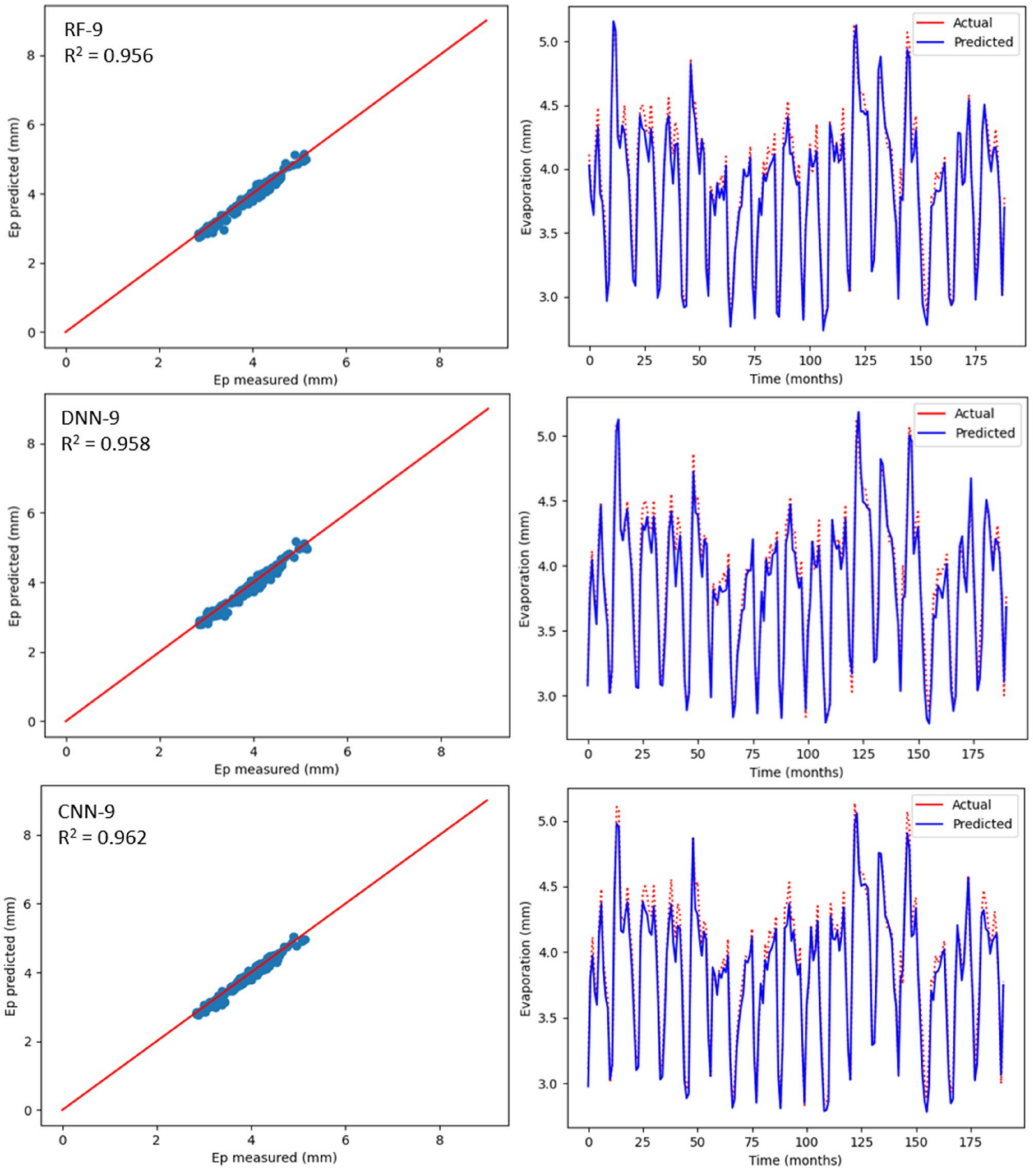
Scatter plot of measured Ep versus predicted Ep for the proposed machine learning models for Kuantan station.

**Figure 16:**
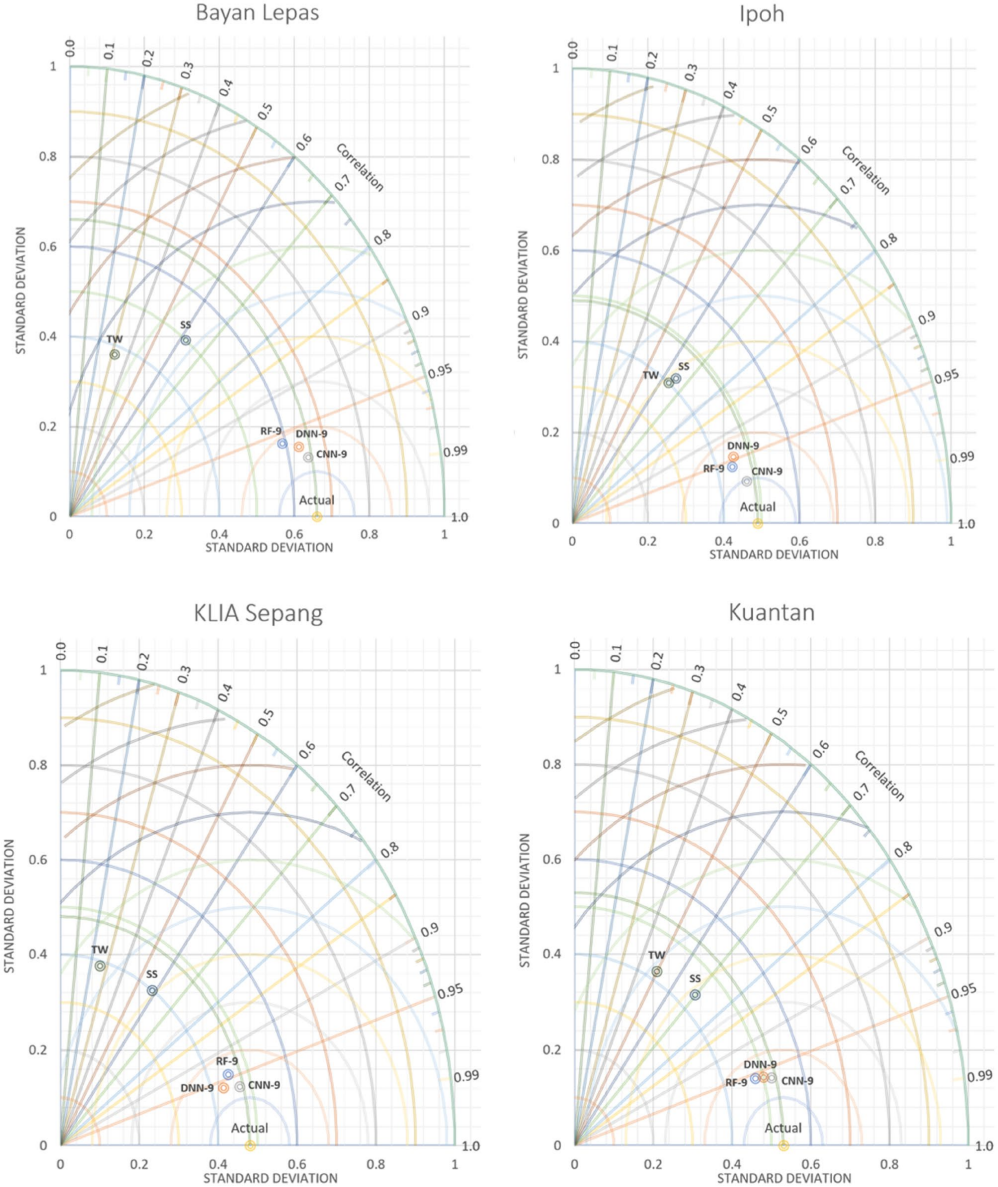
Taylor diagram of predicted monthly pan evaporation during the validation stage.

Table 6 displays the statistical outcomes related to three ML models with the aim to estimate monthly Ep using nine input combinations with respect to meteorological parameters for Bayan Lepas, Ipoh, KLIA Sepang and Kuantan stations. For every ML model, the optimum statistical parameters have been shown in bold. As can be seen in Table 6, there is a noteworthy difference between the estimation accuracy of monthly Ep based on model type and input combination. According to the statistical values, for different input combinations, with respect to the three machine learning models, the CNN-9 model (R^2^ = 0.970, MAE = 0.071, MSE = 0.008, RMSE = 0.092, RAE = 0.138, NSE = 0.980) at the Bayan Lepas station, (R^2^ = 0.980, MAE = 0.053, MSE = 0.004, RMSE = 0.069, RAE = 0.132, NSE = 0.981) at the Ipoh station, (R^2^ = 0.965, MAE = 0.079, MSE = 0.008, RMSE = 0.091, RAE = 0.214, NSE = 0.966) at the KLIA Sepang station, and (R^2^ = 0.962, MAE = 0.084, MSE = 0.010, RMSE = 0.103, RAE = 0.198, NSE = 0.962) at the Kuantan station offered better performance than the DNN and RF models. In addition, as previously stated, the k-fold CV technique has been used. Cross-validation is a reliable method for preventing overfitting. The primary configuration variable for k-fold CV is k, which defines how many folds the dataset will be split into. Hence, as shown in Table 7, different folds (3, 5, and 10) were used in this study. When these k-fold testing values are compared, it is possible to conclude that the CNN model provides the most accurate results with k = 5 for all stations. With the three ML models, estimated values relating to monthly Ep have been plotted against the measured values for each station as shown in Figs. 12, 13, 14 and 15. The lower-level pertaining to scatter plot and an improved fit with respect to the estimated data with that of the values observed in the 1:1 line are the clear indicators suggesting the superiority with respect to the CNN model compared to other models. Even though Figs. 12, 13, 14 and 15 as well as Table 6 display the observed and estimated values for all the models, and also the evaluation criteria, the Taylor diagram (TD) was employed to compare the methods presented in this research. The primary concept of the TD is to represent the closest prediction model with actual corresponding observation in the 2-D scaling (correlation coefficient on polar axis and standard deviation on radial axis). Standard deviation is with respect to how much, on average, measurements vary from each other. Thus, the relative value of SDP from SDA indicates the level of accuracy. The value of SDP from S.D.A. pertains to lower accuracy. Greater difference refers to lower precision. Therefore, in Fig. 16, it can be noticed that the CNN-9 was better compared to other methodologies, which had SD of 0.65 closer to the actual SD of 0.66 in Bayan Lepas, SD of 0.47 to the actual SD of 0.49 in Ipoh, SD of 0.47 to the actual SD of 0.48 in KLIA Sepang, and SD of 0.52 to the actual SD of 0.53 in Kuantan. The comparison of predicted and actual Ep monthly values generated by the most exact models is displayed in Fig. 16, which demonstrated that the ML models are superior to other models generally, while the CNN-9 is superior to the ML models in particular.

**Table 6:** Statistical results (testing period) of the three machine learning models for predicting monthly Ep under nine input combinations of meteorological variables for Bayan Lepas, Ipoh, KLIA Sepang and Kuantan stations.

Station/model	R^2^	MAE	MSE	RMSE	RAE	NSE
**Bayan Lepas**						
RF-1	0.562	0.324	0.195	0.441	0.619	0.563
RF-2	0.727	0.248	0.121	0.348	0.474	0.727
RF-3	0.797	0.219	0.090	0.300	0.418	0.798
RF-4	0.848	0.197	0.066	0.258	0.377	0.849
RF-5	0.857	0.186	0.062	0.250	0.357	0.858
RF-6	0.909	0.149	0.039	0.198	0.288	0.910
RF-7	0.868	0.174	0.057	0.238	0.335	0.869
RF-8	0.953	0.115	0.020	0.142	0.222	0.953
RF-9	**0.961**	**0.088**	**0.016**	**0.128**	**0.169**	**0.962**
DNN-1	0.492	0.351	0.226	0.475	0.671	0.493
DNN-2	0.800	0.224	0.088	0.298	0.429	0.801
DNN-3	0.807	0.223	0.085	0.292	0.427	0.808
DNN-4	0.857	0.188	0.063	0.251	0.359	0.858
DNN-5	0.838	0.197	0.070	0.266	0.378	0.839
DNN-6	0.908	0.147	0.039	0.199	0.283	0.909
DNN-7	0.878	0.158	0.052	0.229	0.304	0.879
DNN-8	0.929	0.135	0.030	0.174	0.260	0.930
DNN-9	**0.969**	**0.087**	**0.012**	**0.113**	**0.169**	**0.970**
CNN-1	0.623	0.290	0.167	0.409	0.554	0.624
CNN-2	0.767	0.231	0.103	0.321	0.442	0.768
CNN-3	0.810	0.222	0.084	0.290	0.425	0.811
CNN-4	0.868	0.178	0.058	0.240	0.341	0.869
CNN-5	0.882	0.167	0.051	0.227	0.321	0.883
CNN-6	0.911	0.148	0.038	0.195	0.286	0.912
CNN-7	0.890	0.174	0.047	0.217	0.335	0.891
CNN-8	0.965	0.084	0.015	0.122	0.163	0.966
CNN-9	**0.979**	**0.071**	**0.008**	**0.092**	**0.138**	**0.980**
**Ipoh**						
RF-1	0.652	0.227	0.084	0.290	0.573	0.653
RF-2	0.737	0.194	0.064	0.253	0.489	0.738
RF-3	0.478	0.291	0.126	0.356	0.735	0.479
RF-4	0.720	0.203	0.068	0.261	0.511	0.721
RF-5	0.734	0.197	0.065	0.256	0.494	0.735
RF-6	0.865	0.140	0.033	0.182	0.348	0.866
RF-7	0.832	0.146	0.041	0.204	0.365	0.833
RF-8	0.901	0.111	0.024	0.157	0.276	0.902
RF-9	**0.959**	**0.066**	**0.010**	**0.101**	**0.163**	**0.960**
DNN-1	0.719	0.205	0.068	0.261	0.519	0.719
DNN-2	0.793	0.162	0.050	0.224	0.408	0.794
DNN-3	0.511	0.277	0.119	0.344	0.701	0.521
DNN-4	0.800	0.173	0.048	0.221	0.436	0.801
DNN-5	0.804	0.170	0.048	0.219	0.424	0.805
DNN-6	0.903	0.123	0.024	0.155	0.307	0.904
DNN-7	0.882	0.122	0.029	0.171	0.305	0.883
DNN-8	0.922	0.106	0.019	0.139	0.264	0.923
DNN-9	**0.945**	**0.094**	**0.013**	**0.116**	**0.232**	**0.946**
CNN-1	0.749	0.192	0.061	0.247	0.486	0.750
CNN-2	0.831	0.152	0.041	0.203	0.382	0.831
CNN-3	0.550	0.270	0.109	0.331	0.683	0.550
CNN-4	0.811	0.171	0.046	0.214	0.430	0.812
CNN-5	0.837	0.157	0.040	0.200	0.393	0.838
CNN-6	0.935	0.096	0.016	0.126	0.239	0.936
CNN-7	0.904	0.113	0.023	0.154	0.281	0.905
CNN-8	0.959	0.071	0.010	0.100	0.176	0.960
CNN-9	**0.980**	**0.053**	**0.004**	**0.069**	**0.132**	**0.981**
**KLIA Sepang**						
RF-1	0.293	0.299	0.167	0.409	0.817	0.294
RF-2	0.730	0.171	0.064	0.253	0.467	0.731
RF-3	0.819	0.077	0.042	0.207	0.212	0.819
RF-4	0.878	0.066	0.028	0.169	0.180	0.879
RF-5	0.880	0.120	0.028	0.168	0.328	0.881
RF-6	0.910	0.108	0.021	0.146	0.293	0.911
RF-7	0.909	0.109	0.021	0.147	0.296	0.910
RF-8	0.931	0.093	0.016	0.128	0.255	0.932
RF-9	**0.943**	**0.070**	**0.013**	**0.117**	**0.190**	**0.944**
DNN-1	0.358	0.287	0.152	0.390	0.783	0.359
DNN-2	0.700	0.183	0.071	0.267	0.499	0.701
DNN-3	0.820	0.141	0.042	0.206	0.385	0.821
DNN-4	0.859	0.129	0.033	0.182	0.355	0.860
DNN-5	0.893	0.059	0.025	0.159	0.161	0.894
DNN-6	0.924	0.101	0.018	0.134	0.275	0.925
DNN-7	0.891	0.119	0.026	0.161	0.324	0.892
DNN-8	0.939	0.100	0.014	0.121	0.269	0.940
DNN-9	**0.959**	**0.071**	**0.009**	**0.099**	**0.191**	**0.960**
CNN-1	0.375	0.284	0.148	0.384	0.774	0.376
CNN-2	0.717	0.178	0.067	0.259	0.485	0.718
CNN-3	0.827	0.079	0.040	0.202	0.216	0.827
CNN-4	0.830	0.137	0.040	0.200	0.375	0.831
CNN-5	0.899	0.114	0.024	0.155	0.313	0.900
CNN-6	0.921	0.101	0.019	0.137	0.274	0.922
CNN-7	0.919	0.101	0.019	0.139	0.274	0.920
CNN-8	0.949	0.081	0.012	0.110	0.219	0.950
CNN-9	**0.965**	**0.079**	**0.008**	**0.091**	**0.214**	**0.966**
**Kuantan**						
RF-1	0.647	0.241	0.098	0.314	0.567	0.648
RF-2	0.811	0.197	0.054	0.232	0.458	0.811
RF-3	0.688	0.237	0.087	0.295	0.556	0.689
RF-4	0.852	0.157	0.041	0.203	0.370	0.853
RF-5	0.888	0.059	0.005	0.074	0.330	0.889
RF-6	0.904	0.122	0.027	0.165	0.283	0.905
RF-7	0.889	0.138	0.031	0.178	0.321	0.890
RF-8	0.930	0.108	0.019	0.140	0.253	0.931
RF-9	**0.956**	**0.088**	**0.012**	**0.110**	**0.207**	**0.957**
DNN-1	0.649	0.248	0.098	0.314	0.581	0.650
DNN-2	0.814	0.180	0.052	0.229	0.422	0.815
DNN-3	0.709	0.228	0.081	0.285	0.573	0.710
DNN-4	0.843	0.166	0.044	0.210	0.389	0.844
DNN-5	0.876	0.146	0.035	0.187	0.339	0.877
DNN-6	0.917	0.121	0.023	0.154	0.282	0.917
DNN-7	0.898	0.132	0.028	0.168	0.309	0.898
DNN-8	0.944	0.103	0.015	0.126	0.238	0.945
DNN-9	**0.958**	**0.084**	**0.011**	**0.108**	**0.198**	**0.959**
CNN-1	0.654	0.239	0.096	0.310	0.562	0.655
CNN-2	0.816	0.187	0.051	0.227	0.437	0.817
CNN-3	0.747	0.210	0.071	0.266	0.491	0.748
CNN-4	0.871	0.149	0.036	0.190	0.350	0.872
CNN-5	0.877	0.143	0.034	0.185	0.335	0.878
CNN-6	0.906	0.124	0.026	0.163	0.287	0.907
CNN-7	0.897	0.130	0.028	0.169	0.304	0.898
CNN-8	0.956	0.086	0.012	0.112	0.198	0.956
CNN-9	**0.962**	**0.084**	**0.010**	**0.103**	**0.198**	**0.962**

Bold indicates the optimum statistical parameters.

**Table 7:** Time series cross-validation.

k-fold CV	R-Squared values			
	Bayan Lepas	Ipoh	KLIA Sepang	Kuantan
K = 3	0.94	0.96	0.95	0.94
K = 5	0.97	0.98	0.96	0.96
K = 10	0.96	0.97	0.95	0.95

As per Table 6, realisation of the best prediction accuracy was possible through the models employing the complete meteorological dataset (T_max_, T_min_, R_s_, S_w_, RH and Ep) with regards to all stations, when compared with combinations pertaining to other incomplete data input. This showed that the model prediction’s accuracy improved in general with additional input parameters, which was similar to the results seen in the earlier studies^3,34^. Four input parameters that have not included R_s_ or S_w_ were adequate to achieve acceptable accuracy with regards to estimation of monthly Ep. When only mean temperature data were available, ML models, including the CNN model, were found to be insufficient for all stations. This implied that employing the powerful capabilities, such as AI may not improve the ML model prediction accuracy, particularly when meteorological inputs are restricted. Besides, with regards to all ML models, the prediction accuracy improved slightly by using Ep as an input. However, the statistical values with regards to machine learning models were close to complete meteorological inputs (i.e., using Ep as an input) by employing the input combination pertaining to T_min_, T_max_, S_w_, R_s_ and RH. This suggested that the estimated monthly Ep values through machine learning models were in general in line with those of the measured monthly Ep values.

Apart from the robustness and convenience associated with DL’s automated feature extraction, it was seen that the proposed deep learning models consistently outdid the RF model when it comes to prediction of Ep. Thus, these research results were in line with the previous studies^53,75^, which mentioned deep learning to be a powerful modelling technique that allows learning the complex and non-linear behaviours pertaining to evaporation. Particularly, it was seen that the CNN model was better than other DLs models, such as DNN, which indicates the CNN model’s high potential when it comes to modelling and mapping evaporation when it is difficult for most of the ML models. The effectiveness pertaining to CNN in capturing and analysing the non-linearity and complexity behaviours of evaporation with greater efficacy could be due to the convolutional characteristic of 1D-CNN, i.e., a large number of convolutional kernels are applied by CNN to the inputs for extracting information extensively, which is helpful for time series forecast. However, DLs versus RFs need to be compared carefully, since there is a chance of underestimating the capacity of RFs when special consideration is not given. Thus, the time needed to run and tune the models also needs to be considered when objectively comparing between DL and RF models. Although training time can be influenced by several factors (e.g., model complexity, number of inputs employed), in general, RF has been found to be faster in tuning and training versus DL. The application of DL includes training time as one of the challenges. In addition to this, it is challenging to optimise DL since no formula has been identified that can guarantee converging of DL to a good solution. Moreover, when compared with the RF, larger data sets are required for DL to learn the evaporation properties. Due to this, even though deep learning is regarded to be very powerful when it comes to capturing complex and non-linear behaviours, there exist certain challenges that need to be taken into account when constructing deep learning prediction models.

With regards to the above statement, the CNN model was seen to be able to model pan evaporation with high prediction accuracy. However, for validating the developed predictive model's predictability, a comparison was performed for the results pertaining to the current study versus other AI models exposed to same climatic conditions. Mustafa et al.^76^ reported an R^2^ value of 0.97 with regards to their best-performing SVM model during validation period by employing the Support Vector Machine (SVM) method in the Ipoh region based on the same data as used in the current research, versus an R^2^ value of 0.98 that was identified in the current study. It was also seen that the CNN model was better compared to other AI methods, including K-Nearest Neighbours (KNN), which was recently used in the Ipoh region based on the same data as employed in the present study (M.A, M.A.I, A.N.A, and Y.F.H). Satisfactory performance was reported by applying the KNN, which gave an R^2^ value of 0.94. Based on this, the study concluded that DL in general, and CNN in particular, can be used as optimistic predictive models in hydrological applications such as evaporation due to the excellent features described earlier. Moreover, investigation will be carried out with regards to the application of the proposed methodology for different regions throughout Malaysia by employing different data sets in order to construct a reliable generalised model for evaporation prediction.

### Comparison of empirical and ML models

Table 8 demonstrates the performances for two empirical models to perform prediction of monthly Ep, which are then compared to their respective ML models using same input combinations for Bayan Lepas, Ipoh, KLIA Sepang and Kuantan weather stations. As an initial observation, with regards to input combination of R_s_ and T_a_ for all stations, the radiation-based model (Stewart and Stephens) offered the lowest prediction accuracy (R^2^ values: 0.620, 0.649, 0.580, and 0.696) in comparison with all ML models. On the other hand, the machine learning models (i.e., RF-1, CNN-1 and DNN-1) were seen to perform excellently to achieve high prediction accuracy versus the temperature-based model (Thornthwaite) based on the input combination of just Ta. Based on the statistical results presented in Table 7, the higher performance of ML models was evident versus empirical models, and could also considerably enhance the prediction accuracy of monthly Ep even when employing the same input parameters, depending on their superior capabilities to carry out non-linear and complex tasks. Furthermore, it has been seen that higher accuracy was achieved with the deep learning models (i.e., DNN and CNN) in terms of forecasting evaporation versus the tree-based model (i.e., RF). This can be attributed to the deep learning feature catching concealed properties, which signifies that deep learning can be regarded as more powerful approach for predicting evaporation. In this regard, although the RF was seen to marginally outperform the DL models for few cases, it is evident that this is a single case since the DL models are regarded to be more consistent and could also offer higher accuracy versus empirical and tree-based methods based on all the different input sets at all stations.

**Table 8:** Statistical results of the empirical and machine learning models under the same input combination for Bayan Lepas, Ipoh, KLIA Sepang and Kuantan weather stations.

Input combination	Station/model	R^2^	MAE	MSE	RMSE	RAE	NSE
	**Bayan Lepas**						
T_a_, R_s_	Stephens & Stewart	0.620	0.328	0.167	0.409	0.631	0.621
	RF-2	0.727	0.248	0.121	0.348	0.474	0.727
	DNN-2	0.800	0.224	0.088	0.298	0.429	0.801
	CNN-2	0.767	0.231	0.103	0.321	0.442	0.768
T_a_	Thornthwaite	0.317	0.431	0.306	0.553	0.820	0.317
	RF-1	0.562	0.324	0.195	0.441	0.619	0.563
	DNN-1	0.492	0.351	0.226	0.475	0.671	0.493
	CNN-1	0.623	0.290	0.167	0.409	0.554	0.624
	**Ipoh**						
T_a_, R_s_	Stephens & Stewart	0.649	0.231	0.085	0.292	0.585	0.650
	RF-2	0.737	0.194	0.064	0.253	0.489	0.738
	DNN-2	0.793	0.162	0.050	0.224	0.408	0.794
	CNN-2	0.831	0.152	0.041	0.203	0.382	0.831
T_a_	Thornthwaite	0.635	0.243	0.088	0.296	0.615	0.636
	RF-1	0.652	0.227	0.084	0.290	0.573	0.653
	DNN-1	0.719	0.205	0.068	0.261	0.519	0.719
	CNN-1	0.749	0.192	0.061	0.247	0.486	0.750
	**KLIA Sepang**						
T_a_, R_s_	Stephens & Stewart	0.580	0.244	0.098	0.314	0.670	0.581
	RF-2	0.730	0.171	0.064	0.253	0.467	0.731
	DNN-2	0.700	0.183	0.071	0.267	0.499	0.701
	CNN-2	0.717	0.178	0.067	0.259	0.485	0.718
T_a_	Thornthwaite	0.256	0.325	0.175	0.418	0.891	0.257
	RF-1	0.293	0.299	0.167	0.409	0.817	0.294
	DNN-1	0.358	0.287	0.152	0.390	0.783	0.359
	CNN-1	0.375	0.284	0.148	0.384	0.774	0.376
	**Kuantan**						
T_a_, R_s_	Stephens & Stewart	0.696	0.245	0.085	0.292	0.572	0.697
	RF-2	0.811	0.197	0.054	0.232	0.458	0.811
	DNN-2	0.814	0.180	0.052	0.229	0.422	0.815
	CNN-2	0.816	0.187	0.051	0.227	0.437	0.817
T_a_	Thornthwaite	0.497	0.284	0.144	0.380	0.657	0.498
	RF-1	0.647	0.241	0.098	0.314	0.567	0.648
	DNN-1	0.649	0.248	0.098	0.314	0.581	0.650
	CNN-1	0.654	0.239	0.096	0.310	0.562	0.655

## Conclusion

This study is conducted to determine the monthly Ep losses by employing RF, DNN, and CNN techniques. Monthly data from four weather stations in Malaysia were employed to assess the capabilities of the three AI approaches in predicting the Ep rates. Time series data pertaining to monthly Ep, such as T_max_, T_min_, T_a_, RH, S_w_, R_s_, and E_p_, between the years 2000–2019 were used to set up the evaluated models. The data was divided into two parts: 20% for testing (validation) and 80% for training (calibration). The PCC values were used to select the input parameters (predictors) in order to identify the most effective input combinations for ML models. The developed ML models were compared to two empirical models, one is temperature-based model (Thornthwaite) while the other is radiation-based model (Stephens & Stewart). Standard statistical measures were employed to assess the performance of each model as well as their effectiveness pertaining to evaporation forecasting. Furthermore, the accuracy of the studied models was evaluated using the Taylor diagram. The investigation yielded the following results:

- The three developed ML models were found to outperform the empirical methods and to significantly improve the precision of monthly Ep estimates even when using the same combinations of inputs.
- Both RF and DL methods can accurately predict the monthly Ep. In particular, when it comes to predicting Ep, the DL approach (i.e., CNN and DNN) was found to slightly outperform the RF model.
- The best ML prediction accuracy could be achieved with models that employed complete meteorological datasets (T_max_, T_min_, R_s_, S_w_, RH and Ep) with regards to all stations, when compared with other combinations of incomplete data input.
- As seen in the results, the monthly evaporation losses can be successfully modelled based on the CNN structure along with enhanced accuracy versus other models that were accounted in this study. Moreover, estimation results based on the CNN model were seen to outdo versus other AI approaches that were studied in the same regions by employing the same data.
- In the future, the applicability of the proposed methodology to different regions in Malaysia can be assessed using different data sets with the aim of building a dependable generalised model for predicting evaporation.

## Data Availability

The datasets used during the current study are available from the first author on reasonable request.

## Abbreviations

AI: Artificial intelligence
ANN: Artificial neural networks
CNN: Convolutional neural network
CV: Cross-validation
DL: Deep learning
DNN: Deep neural network
Ep: Pan evaporation
MAE: Mean absolute error
ML: Machine learning
MSE: Mean square error
NSE: Nash–Sutcliffe efficiency
PCC: Pearson correlation coefficient
R^2^: Coefficient of determination
RAE: Relative absolute error
RF: Random Forest
RH: Relative humidity
RMSE: Root mean square error
R_s_: Solar radiation
SD: Standard deviation
SDA: Standard deviation actual
SDP: Standard deviation predicted
S_w_: Wind speed
T_a_: Mean air temperature
T_max_: Maximum air temperature
T_min_: Minimum air temperature
